# Thermal comfort enhancement in Oum El Bouaghi (Algeria) using PCM-enhanced walls and natural ventilation: a comparative CFD study

**DOI:** 10.1038/s41598-026-43308-y

**Published:** 2026-03-25

**Authors:** Moussa Aidi, Yamina Harnane, Müslüm Arıcı, Lyes Bordja, Tibor Poós

**Affiliations:** 1 Department of Mechanical Engineering, Faculty of Sciences and Applied Sciences, University of Larbi Ben Mhidi, Oum El Bouaghi, Algeria; 2 Laboratory of Advanced Design and Modeling of Mechanical Systems and Thermo-Fluid (CMASMTF), University of Larbi Ben Mhidi, Oum El Bouaghi, Algeria; 3 Mechanical Engineering Department, Engineering Faculty, Kocaeli University, Kabaoğlu, Umuttepe Yerleşkesi, İzmit/Kocaeli, 41001 Turkey; 4 Department of Building Services and Process Engineering, Faculty of Mechanical Engineering, Budapest University of Technology and Economics, Műegyetem rkp. 3, Budapest, H-1111 Hungary

**Keywords:** Phase change material, Ventilation strategies, Thermal comfort, Effective draft temperature, Synergy, Energy science and technology, Engineering

## Abstract

This study presents a comparative parametric investigation of the combined effect of phase change materials (PCMs) integrated into brick walls and natural ventilation strategies on indoor thermal comfort under the arid summer climate of Oum El Bouaghi, Algeria. A CFD analysis using ANSYS Fluent evaluated four PCMs (hexahydrate, n-hexadecane, n-eicosane, and n-octadecane) and three ventilation configurations with different inlet–outlet arrangements. The PCM performance was analyzed over seven consecutive July days using measured outdoor temperatures as boundary conditions, while ventilation scenarios were assessed during representative hot days to reduce computational cost. Thermal performance was assessed through indoor air temperature, wall heat flux, effective draft temperature (EDT), and the field synergy angle between velocity and temperature-gradient vectors. Although n-hexadecane exhibited the highest instantaneous heat absorption and the lowest internal surface temperatures during peak periods, its early phase transition limited sustained thermal regulation. In contrast, n-octadecane, with a melting range of 301–302 K, provided more stable and prolonged temperature control, making it the most suitable PCM among the investigated candidates when considering both thermal stability and indicative material cost. A parametric thickness analysis showed that increasing PCM thickness up to 10–15 cm led to diminishing thermal returns, achieving up to 52% reduction in daily integrated heat flux compared to the brick-only reference wall under July conditions. However, this range represents an upper-bound performance scenario; from an engineering feasibility perspective, thinner PCM layers (e.g., 5–10 cm) may provide a more practical balance between constructability and thermal benefit. The ventilation configuration with a bottom inlet and top outlet on opposite walls yielded the most stable indoor conditions due to improved air circulation. The study also introduces EDT and a synergy parameter to quantify the interaction between heat transfer and ventilation. Overall, combining PCM-enhanced walls with climate-adaptive ventilation demonstrates significant potential for reducing cooling demand and improving sustainable building performance in hot climates.

## Introduction

Buildings account for a large share of global energy consumption and greenhouse gas emissions, reaching about 45% worldwide and nearly 40% in the European Union, highlighting the urgent need to improve energy efficiency and reduce dependence on fossil fuels^1,2^. In this context, phase change materials (PCMs) have gained increasing attention as effective thermal energy storage solutions for buildings, particularly when integrated into the building envelope as passive systems to enhance thermal regulation and indoor comfort^3^. Recent advances in PCM technologies, including nanoparticle-enhanced and smart PCMs, have further improved thermal performance and expanded their applications beyond buildings to areas such as energy storage, electronics cooling, and medical systems^4–6^. Experimental studies have demonstrated that PCM integration can significantly reduce indoor temperature fluctuations and increase thermal delay, confirming their potential for sustainable and energy-efficient building applications^7–18^ active systems, which require additional energy input, can contribute to reducing overall energy consumption when properly designed, while effective ventilation strategies including passive ventilation play a crucial role in lowering energy demand by reducing reliance on mechanical heating and cooling equipment. Passive ventilation focuses on supplying cool air with minimal electricity consumption and thus serves as an alternative to energy-intensive mechanical air-conditioning systems^15,19^. Comparative results reported in^15^ indicate that free cooling combined with PCM is more effective than passive PCM applications alone in reducing indoor temperatures. Specifically, free cooling can lower indoor air temperature by up to 1.8 K, whereas passive thermal storage achieves a reduction of approximately 0.5 K. Consequently, combining passive ventilation with PCM-based energy storage represents a promising strategy for achieving substantial energy savings^20^ Performed both experimental and numerical analyses of a latent heat storage unit for heating and cooling, employing 30 paraffin-filled RT22HC plates and monitoring the thermal response to temperature variations. Annual simulations showed that the thermal energy storage (TES) system provided maximum heating in March and maximum cooling storage in July and August, resulting in an annual energy saving of approximately 142 kWh for an office building. Another study^21^ demonstrated the feasibility of free cooling under unfavorable climatic conditions by proposing and optimizing a new system incorporating PCM-based energy storage. Using building energy simulation (BES), energy consumption and occupant comfort were evaluated, and the optimized system achieved a 36% reduction in cooling energy demand and a 35% improvement in thermal comfort. a comprehensive review by^22^examined the use of PCMs in building envelopes, emphasizing their ability to reduce cooling loads and shift peak energy demand, thereby improving energy efficiency and occupant comfort. The study reviewed different PCM types (e.g., organic and inorganic) and assessed their effectiveness in building walls, highlighting that PCM encapsulation enhances durability and compatibility with conventional construction materials, which is essential for long-term applications. Previous studies^22^on PCM-reinforced walls have primarily focused on extreme climatic conditions, analyzing indoor temperature stability and demonstrating that selecting a PCM melting temperature tailored to seasonal variations optimizes performance for passive cooling in hot climates and heat storage during colder periods. enard et al.^23^analyzed energy savings achieved through improved night ventilation in 14 office rooms in low-energy buildings, demonstrating that free cooling potential can be estimated using thermal comfort limits and free operating temperature differences. Studies reported in^24–26^ investigated the charging and discharging processes in free cooling systems for hot and dry climates, identifying the PCM melting temperatures of approximately 302 K in summer, 294 K in winter, and 300.05 K for year-round operation. Darzi et al.^27^ examined the effects of heat transfer fluid (HTF) mass flow rate, inlet temperature, PCM plate thickness, and melting front evolution on cooling power, showing that higher mass flow rates enhance cooling capacity, while PCM plate thickness exhibits a linear relationship with melting duration. Additionally, an experimental study^28^ evaluated the free cooling potential of inorganic PCMs for daytime temperature reduction, achieving an average indoor temperature decrease of 2.5 K and suggesting that further reductions could be achieved by minimizing thermal losses. despite extensive research on PCMs and natural ventilation as independent strategies, a comprehensive investigation of their combined application remains limited. The Nat Vent project^29^ identified insufficient expertise and limited knowledge among stakeholders as major barriers to the adoption of natural ventilation systems. In low-rise buildings during summer, wind-driven airflow becomes a dominant mechanism, motivating ventilation strategies that harness this natural phenomenon. Strategically placed openings in the building envelope facilitate airflow, and study^30^ highlighted the potential of wind-driven natural ventilation using wind towers to enhance energy efficiency and thermal comfort in hot-climate, low-rise buildings. Wind towers employ passive cooling and natural airflow to reduce cooling loads, minimize dependence on mechanical systems, and maintain adequate ventilation rates. Marzieh et al.^31^ investigated the impact of wind-driven natural ventilation on indoor air quality and air exchange rates in office environments. Using a validated computational fluid dynamics (CFD) model, approximately 150 cases of single-sided (SV) and double-sided (DV) ventilation configurations were analyzed, focusing on airflow patterns and geometric parameters. The results identified five distinct airflow patterns in DV cases and demonstrated the critical influence of airflow velocity and room dimensions on air changes per hour (ACH). In SV configurations, occupant location and airflow angle were found to significantly affect ventilation performance. These findings emphasize the importance of wind-driven ventilation in improving indoor air quality, productivity, and occupant well-being. Yamanaka et al.^32^ experimentally evaluated two models for wind-driven unilateral ventilation: pulsation theory (Cockcroft and Robertson^33^) and mixing layer theory (Warren^34,35^). Research on buildings with multiple openings includes the work of Linden et al.^35^ on buoyancy-driven ventilation, which forms the foundation of many natural ventilation models. Chen et al.^36^ proposed a multilayer model for displacement ventilation in single-zone buildings, validated through water-bath experiments using microbubble techniques. Fitzgerald and Woods^37^ studied natural ventilation in rooms with central heating and ventilation openings at different heights, demonstrating that increasing the vertical distance between openings enhances overall ventilation flow, particularly when upper and lower vents are widely separated.

Previous studies have extensively examined phase change materials (PCMs) and natural ventilation as independent strategies for improving building performance; however, their combined potential remains largely underexplored, particularly in terms of predictive thermal comfort parameters, material placement, and realistic occupant comfort assessment under diverse climatic conditions. The present work is a parametric investigation rather than a formal optimization study, aiming to systematically evaluate the influence of key design and operating parameters on thermal performance and comfort outcomes. Specifically, this study investigates the integration of PCMs and natural ventilation within building walls under the climatic conditions of Oum El Bouaghi, Algeria. Four PCMs—hexahydrate, n-hexadecane, n-eicosane, and n-octadecane—are comparatively assessed to identify the most effective material, as well as suitable placement and layer thickness, based on performance comparison rather than the minimization of a predefined objective function.

A key contribution of this work is the introduction of two novel thermal comfort indicators: the effective draft temperature (EDT) and a synergy parameter, which together provide deeper insight into the coupled effects of airflow velocity and heat transfer in defining comfort zones. In addition, a detailed parametric analysis of ventilation configurations, accounting for prevailing wind conditions in Oum El Bouaghi, enables the identification of favorable inlet and outlet orientations.

## Numerical model

### Physical model

**Fig. 1:**
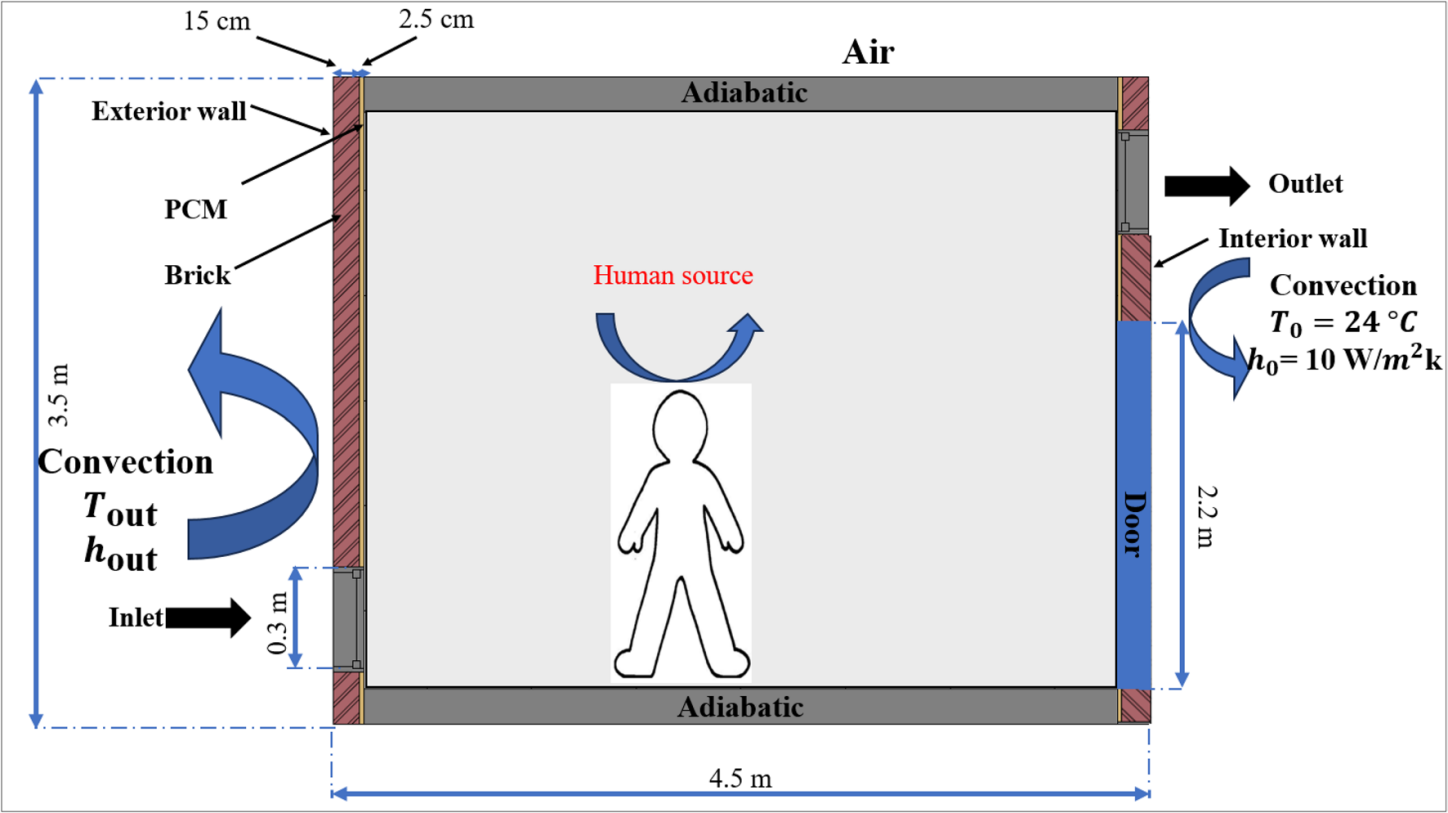
A schematic drawing of the study room, a schematic diagram defining all the boundary conditions.

**Fig. 2:**
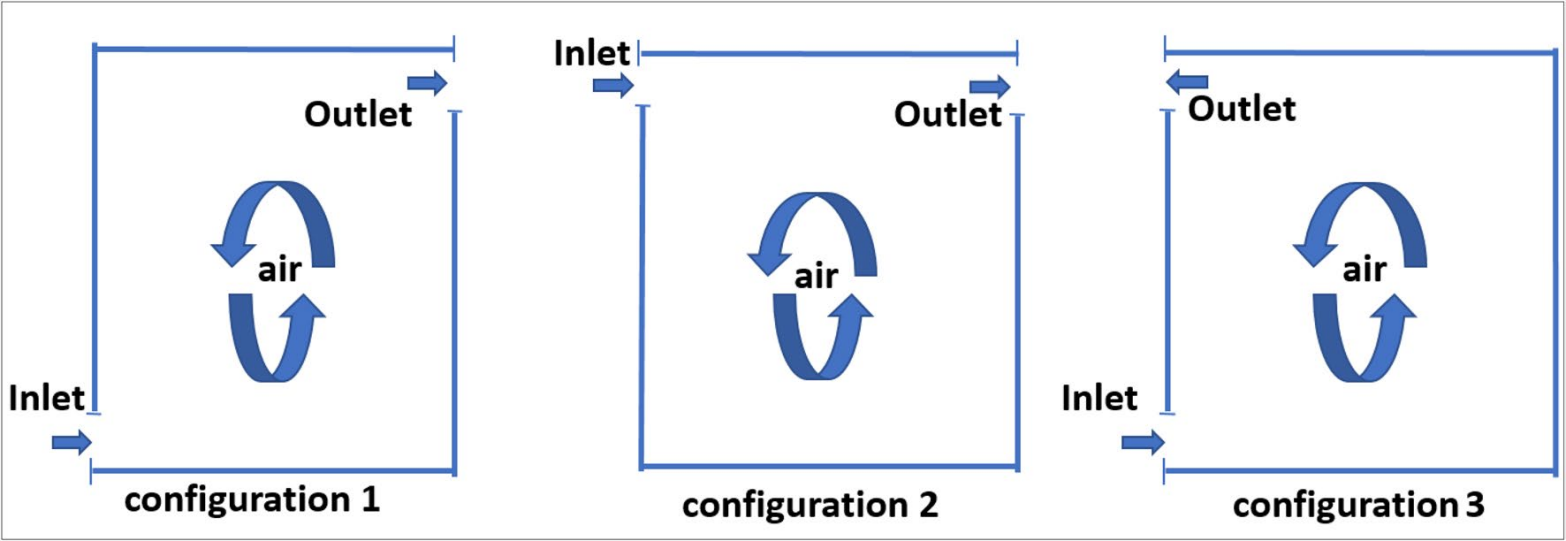
A schematic drawing of the study room, a description of the study configurations.

**Fig. 3:**
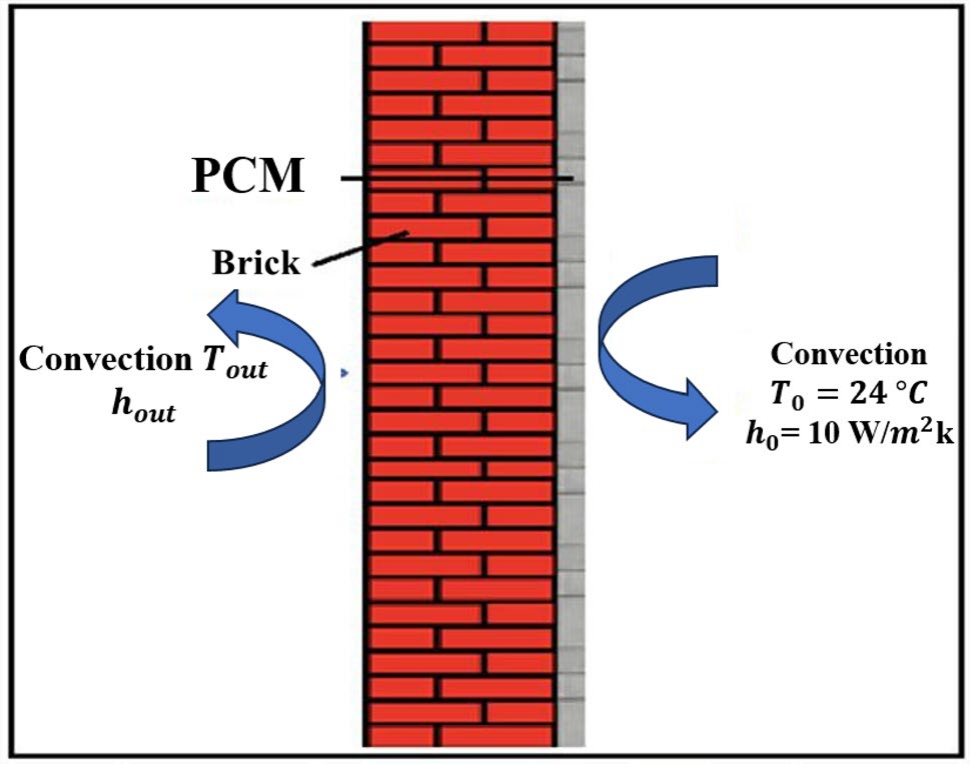
The defined wall of the room.

Figure 1 shows a schematic diagram of the enclosed room, delineating all boundary conditions and describing the configurations designed for the study. The room is modeled as a rectangular cavity measuring 3.5 meters in height and 4.5 meters in width, featuring two air inlet and outlet openings, each 0.3 meters long. A rectangular shape at the center represents an occupant and a computer, both acting as heat sources; the occupant is assumed to occupy the room from 8 a.m. to 12 p.m. and from 1 p.m. to 5 p.m., simulating heat emissions. The left wall contains a layer of phase change material (PCM) exposed to external heat, while the right wall is subjected to internal building heat. The ceiling and floor are insulated as illustrated in Figure 1. Figure 2 presents the selected ventilation configurations: the first with two openings on opposite walls, one near the floor and one near the ceiling; the second with two openings on opposite walls, both located near the ceiling; and the third with two openings on the same wall near the floor and ceiling. Figure 3 shows the selected wall, which was studied independently to identify the most appropriate phase change material (PCM) type, as well as its most effective placement and thickness under the investigated boundary conditions. The PCM layer is assumed to be encapsulated between two thin aluminum sheets. Due to aluminum’s high thermal conductivity and the small thickness of the sheets, their thermal resistance is neglected, and the wall is modeled as a brick–PCM composite with ideal contact. Table 1 lists the physical properties of all materials used.

**Table 1:** Main thermophysical parameters of the used materials^38–40^.

Properties of the materials	(Hexa-Hydrate)	n-hexadecane	n-eicosane	n-octadecane	Unfilled brick
Density, (kg/${{\boldsymbol{m}}}^{3}$)	1802 (Solid) 1562 (Liquid)	$-0.6854\mathrm{T}+787.2$	$-0.549\mathrm{T}+945.722$	$-0.65\mathrm{T}+971.5212$	1600
Thermal conductivity, $(\mathrm{W}/(\mathrm{mK}))$	1,09 (Solid296.15 $\mathrm{K}$)/ 0,54 (Liquid 311.85 $\mathrm{K}$)	$278.15<\mathrm{T}<290.15\,\lambda =0.341$ $293.15<\mathrm{T}<313.15\,\lambda =0.150$	$260<\mathrm{T}<290\lambda =0.425$ $310<\mathrm{T}<3505\lambda =0.15$	$290<\mathrm{T}<295\lambda =0.33$ $301<\mathrm{T}<350\lambda =0.15$	0.7
Specific heat,${{\boldsymbol{c}}}_{{\boldsymbol{p}}},(\mathrm{J}/\mathrm{kgK})$	1400 (Solid 297.15 $\mathrm{K}$)/2310 (Liquid 305.15 $\mathrm{K}$)	2240/4000	$$\begin{aligned} & \left( {{\mathrm{Solid}}} \right) \\ & 1.048 \cdot 10^{ - 6} T^{4} - 1.165 \cdot 10^{ - 3} T^{3} \\ & + 0.485T^{2} + - 89.957T + 6245 \hfill \\ \end{aligned}$$ $$\begin{aligned} &\left( {{\mathrm{Liquid}}} \right) \\ &3.192 \cdot 10^{ - 7} T^{4} - 4.188 \cdot 10^{ - 4} T^{3} \\ &+ 0.206T^{2} - 45.041T + 3694 \\ \end{aligned}$$	$$\begin{aligned} &\left( {{\mathrm{Solid}}} \right) \\ &8.804 \cdot 10^{ - 7} T^{4} - 9.390 \cdot 10^{ - 4} T^{3} \\ &+ 0.375T^{2} - 66.573T + 4425 \\ \end{aligned}$$ $$\begin{aligned} &\left( {{\mathrm{Liquid}}} \right) \\& 1.698{ } \cdot 10^{ - 7} T^{4} - 2.229 \cdot 10^{ - 4} T^{3} \\& + 0.1098T^{2} - 24.026T + 1975 \\ \end{aligned}$$	840
Melting temperature (${{\boldsymbol{T}}}_{solid };{{\boldsymbol{T}}}_{liquid }),$ (K)	302.15	288.65; 290.9	307.15	301.15	-
Latent Heat $(\mathrm{J}/\mathrm{Kg})$ (at atmospheric pressure)	188,34	235,95	248,00	244,00	-

Computational fluid dynamics (CFD) simulations were performed using ANSYS Fluent, a certified building simulation tool known for its advanced numerical modeling capabilities. The simulations incorporated functions to represent external temperature variations, air velocity, and indoor temperature, as well as a heat source function representing the occupant and computer, linked to an external file providing real-time ambient temperature data over time. The study focuses on Oum El Bouaghi, Algeria, specifically examining two representative hot days in July for ventilation configuration testing, chosen to reflect typical weekly conditions and to optimize computational efficiency. A limitation of the present work is the focus on July (cooling-dominant conditions); PCM effectiveness is climate- and season-dependent, and therefore, annual simulations including winter and shoulder seasons are required to generalize the conclusions. The PCM-integrated walls were simulated over seven days to determine the best PCM type, thickness, and placement within the wall. This study concentrates on passive strategies combining PCM and natural ventilation with no explicit HVAC system modeled; HVAC is referenced only to contextualize potential reductions in mechanical cooling demand.

The PCM layer is applied uniformly over the entire surface area of the selected wall (Figure 3). Therefore, the total PCM volume ${V}_{\mathrm{PCM}}$ is calculated as:

1 $${V}_{\mathrm{PCM}}={A}_{\mathrm{wall}}\times {th}_{\mathrm{PCM}}$$

where ${A}_{\mathrm{wall}}$ is the wall surface area and ${th}_{\mathrm{PCM}}$ is the PCM thickness, ranging from 2.5 cm to 15 cm depending on the case.

### Mathematical formulation

To validate the accuracy of the simulated airflow within the square cavity, a set of assumptions has been established. These assumptions encompass various aspects of the airflow dynamics:

- The air is considered Newtonian and incompressible.
- Both viscous dissipation and heat transfer by radiation are negligible.
- The airflow inside the cavity is considered turbulent and unstable.
- Thermophysical properties of air are presumed to be constant, except for conditions related to buoyant forces.
- The fusion of PCM is considered unstable and occurs in a two-dimensional manner.
- The PCM is assumed to be both homogeneous and isotropic.
- The movement of PCM in the liquid state is assumed to be laminar.

Under these assumptions, the equations describing the airflow inside the square cavity are formulated as follows:

### Governing equations

The governing equations for mixed convection in an open cavity include the continuity equation, the momentum equation, and the energy equation. These equations, expressed in tensor form within the Cartesian coordinate system, can be written as follows:

Continuity equation**:**

2 $$\frac{\partial \rho }{\partial \mathrm{t}}+\frac{\partial }{\partial {\mathrm{x}}_{\mathrm{i}}}\left(\rho {\mathrm{u}}_{\mathrm{i}}\right)=0$$

Momentum equation:

3 $$\frac{\partial }{\partial \mathrm{t}}\left(\rho {\mathrm{u}}_{\mathrm{i}}\right)+\frac{\partial }{\partial {\mathrm{x}}_{\mathrm{j}}}\left(\rho {\mathrm{u}}_{\mathrm{i}}{\mathrm{u}}_{\mathrm{j}}\right)=-\frac{\partial \mathrm{p}}{\partial {\mathrm{x}}_{\mathrm{i}}}+\frac{\partial }{\partial {\mathrm{x}}_{\mathrm{j}}}\left[\mu \left(\frac{\partial {\mathrm{u}}_{\mathrm{i}}}{\partial {\mathrm{x}}_{\mathrm{j}}}+\frac{\partial {\mathrm{u}}_{\mathrm{j}}}{\partial {\mathrm{x}}_{\mathrm{i}}}\right)-\rho \overline{{\mathrm{u} }_{\mathrm{i}}^{\rm{^{\prime}}}{\mathrm{u}}_{\mathrm{j}}^{\rm{^{\prime}}}}\right]-\rho \overrightarrow{\mathrm{g}}\beta \left(\mathrm{T}-{\mathrm{T}}_{\mathrm{ref}}\right){\hat{\mathrm{e}}}_{i}$$

Energy equation for the air in the cavity:

Boundary condition for external wall (with PCM Layer):

4 $${\mathrm{q}}_{\mathrm{out}}={\mathrm{h}}_{\mathrm{out}}({\mathrm{T}}_{\mathrm{OUT}}-{\mathrm{T}}_{\mathrm{wall}})$$

Boundary condition for internal wall:

5 $${\mathrm{q}}_{\mathrm{in}}={\mathrm{h}}_{0}({\mathrm{T}}_{0}-{\mathrm{T}}_{\mathrm{wall}})$$

6 $$\frac{\partial }{\partial \mathrm{t}}(\rho \mathrm{T})+\frac{\partial }{\partial {x}_{j}}\left(\rho {u}_{j}T\right)=\frac{\partial }{\partial {\mathrm{x}}_{\mathrm{j}}}\left[\frac{\mu }{\mathrm{Pr}}\frac{\partial \mathrm{T}}{\partial {\mathrm{x}}_{\mathrm{j}}}-\rho \overline{{\mathrm{u} }_{\mathrm{j}}^{\rm{^{\prime}}}{\mathrm{T}}^{\rm{^{\prime}}}}\right]$$

Based on the previous assumptions, it was done the Reynolds stress tensor $-\rho \overline{{u }_{1}{\prime}{u}_{\rm{j}}{\prime}}$ and the turbulent heat flux $-\rho {\mathrm{u}}^{\prime}_{\mathrm{j}}\mathrm{Tju}^{\prime}$ were modeled through Boussinesq assumption as follows:

7 $$-\rho \overline{{u }_{i}^{\rm{^{\prime}}}{\mathrm{u}}_{\mathrm{j}}^{\rm{^{\prime}}}}={\mu }_{\mathrm{t}}\left(\frac{\partial {\mathrm{u}}_{\mathrm{i}}}{\partial {\mathrm{x}}_{\mathrm{j}}}+\frac{\partial {\mathrm{u}}_{\mathrm{j}}}{\partial {\mathrm{x}}_{\mathrm{i}}}\right)-\frac{2}{3}\left(\rho \mathrm{k}+{\mu }_{\mathrm{t}}\right)$$

8 $$-\rho \overline{{u }_{j}{\prime}{T}{\prime}}=\frac{{\mu }_{\mathrm{t}}}{{\mathrm{Pr}}_{\mathrm{t}}}\frac{\partial \mathrm{T}}{\partial {\mathrm{x}}_{j}}$$

To further close the equations, the RNG $k-\varepsilon$ turbulent model was adopted in this study, which includes the following equation to obtain ${\mu }_{\mathrm{t}}$ and $k$.

9 $${\mu }_{t}=\rho {\mathrm{C}}_{\mu }\frac{{\mathrm{k}}^{2}}{\varepsilon }$$

10 $$\frac{\partial }{\partial t}(\rho k)+\frac{\partial }{\partial {x}_{i}}\left(\rho k{u}_{i}\right)=\frac{\partial }{\partial {x}_{j}}\left[\left(\mu +\frac{{\mu }_{t}}{{\sigma }_{k}}\right)\frac{\partial k}{\partial {x}_{i}}\right]+{G}_{k}+{G}_{b}-\rho \varepsilon$$

11 $$\frac{\partial }{\partial t}(\rho \varepsilon )+\frac{\partial }{\partial {\mathrm{x}}_{\mathrm{i}}}\left(\rho \varepsilon {\mathrm{u}}_{\mathrm{i}}\right)=\frac{\partial }{\partial {\mathrm{x}}_{\mathrm{j}}}\left[\left(\mu +\frac{{\mu }_{t}}{{\sigma }_{\varepsilon }}\right)\frac{\partial \varepsilon }{\partial {\mathrm{x}}_{\mathrm{j}}}\right]+{\mathrm{C}}_{1\varepsilon }\frac{\varepsilon }{\mathrm{k}}\left({\mathrm{G}}_{\mathrm{k}}+{\mathrm{C}}_{3\varepsilon }{\mathrm{G}}_{\mathrm{b}}\right)-{\mathrm{C}}_{2\upvarepsilon }\rho \frac{{\varepsilon }^{2}}{\mathrm{k}}$$

where ${\mathrm{G}}_{\mathrm{k}}$ the producing is shear rate of the turbulent kinetic energy and ${\mathrm{G}}_{\mathrm{b}}$ is the buoyancy generation rate of the turbulent kinetic energy. Other model constants can be found in the reference^41^.

In this study, the energy equation for the wall containing PCM is adopted separately The final approach to addressing the thermal aspects of the problem, known as the total enthalpy formulation, involves expressing all terms of the energy equation in terms of the mass enthalpy *ℎ* of the PCM, thereby eliminating the temperature variable. The following equation has been used in the calculations^42^:

12 $$\rho \frac{\partial {H}_{\mathrm{sens} \, }(T)}{\partial t}+\nabla {H}_{\mathrm{sens} \, }(T)\rho \overrightarrow{v}=\nabla (\uplambda \nabla T)-\rho \left(\frac{d{H}_{lat \, }}{dt}+\overrightarrow{v}\overrightarrow{\nabla }{H}_{lat \, }\right)+{\mathrm{h}}_{\mathrm{out}}({\mathrm{T}}_{\mathrm{OUT}}-{\mathrm{T}}_{\mathrm{wall}})+{\mathrm{h}}_{0}({\mathrm{T}}_{0}-{\mathrm{T}}_{\mathrm{wall}}$$

In the energy equation, the sensible and latent enthalpies are expressed as follow^42^:

13 $${H}_{sens }(T)={h}_{ref }+{\int }_{{T}_{m}}^{T} {c}_{P}dT$$

14 $${H}_{lat }(T)=f(T)\cdot \Delta {H}_{S-L}$$

External convection (outdoor surface). The external convective heat transfer coefficient at the outer wall is estimated using the full-scale façade correlation proposed by Loveday and Taki (1996)^43^ for a smooth vertical surface on a multi-storey building façade:.

15 $${h}_{out}=2.00 \mathrm{w}+8.91 .$$

where ${h}_{out}$ is the wind speed used in the present study. This expression corresponds to the windward regression reported by Loveday and Taki (1996) (their Eq. 15), originally correlated with roof/free-stream wind speed measured above the roof level. In the current work, *w* denotes the wind speed provided by the local meteorological dataset used to prescribe the outdoor boundary conditions; the July wind speeds in our input data fall within approximately 0–5 m/s which is the effective range applied in our simulations. Because outdoor convection correlations for buildings are empirical and site-dependent, the resulting ${h}_{out}$ carries uncertainty. For building-envelope simulations, uncertainties on the order of ±10–20% are commonly expected for forced-convection estimates. This uncertainty may influence the absolute heat-flux magnitude; however, since the same ${h}_{out}$(w) formulation is applied consistently across all PCM cases, it does not affect the comparative ranking of the investigated configurations.

### Synergy angle

The study of heat transfer is a fundamental aspect of mechanical engineering. This article begins by reassessing the mechanism of convective heat transfer. It highlights the inadequacy of existing parameters for determining thermal comfort zones, leading to the proposal of a new parameter, the synergy parameter, derived from mixer reviews. In the field of mixers, heat transfer rates depend not only on flow surfaces and temperatures, but also on their synergy, as shown by Z.-Y. Guo et al.^44^ in 2005. The article by^45^ Xiaohuan Zhao et al The results of a theoretical and computational study are presented, aiming to directly apply the field synergy principle to enhance heat transfer and to identify configurations that provide improved thermal comfort in buildings. The research extends field synergy theory to encompass desirable attributes for various applications, providing a conceptual framework and research methodology for advancing thermal energy and conversion. As previously mentioned, synergy has been used in mixer magazines^46^.Fang Li et al., 2019 Using a 3D numerical simulation of a conjugate heat transfer model, this work investigates the field synergy and heat transfer performance of microchannels with three different cavity configurations. Synergistic field angle, Nu number and cavity direction are found to have a significant constant relationship in predicting heat transfer enhancement in terms of internal cavities in the microchannel. The heat transfer enhancement of nanofluids in this complex microchanne l was studied by analyzing the field synergy angle. To study heat transfer, it is necessary to know the phenomenon of field synergy. This phenomenon can be briefly demonstrated by the energy equation^47,48^ (Z.Y. Guo et al., 1998; Tao et al., 2002).

16 $$\theta =\mathrm{arccos}\left(\frac{U\frac{\partial T}{\partial X}+V\frac{\partial T}{\partial Y}}{\sqrt{{U}^{2}+{V}^{2}}\sqrt{{\left(\frac{\partial T}{\partial X}\right)}^{2}+{\left(\frac{\partial T}{\partial Y}\right)}^{2}}}\right)$$

Research is focusing on synergistic multi-domain relationships between velocity, pressure, and temperature and component concentration. Synergistic equations, based on the conservation of thermal energy, mechanical energy, component mass and fluid momentum, reveal thermophysical properties.

### Effective draft temperature

Effective draft temperature (EDT) Created by Rydberg and Norback^49^ then modified by Koestel and Tuve^50^ is one of the primary thermal indices. It combines temperature and air speed. Effective draft temperature values varying between $-1.7<EDT<1.1$ characterize thermal comfort while EDT values outside this range represent the thermal discomfort zone. According to Koestel and Tuve^50^, the effective draft temperature is defined as:

17 $$EDT=\left({T}_{x}-{T}_{m}\right)-8({V}_{x}-0.15)$$

## Validation and verification

**Fig. 4:**
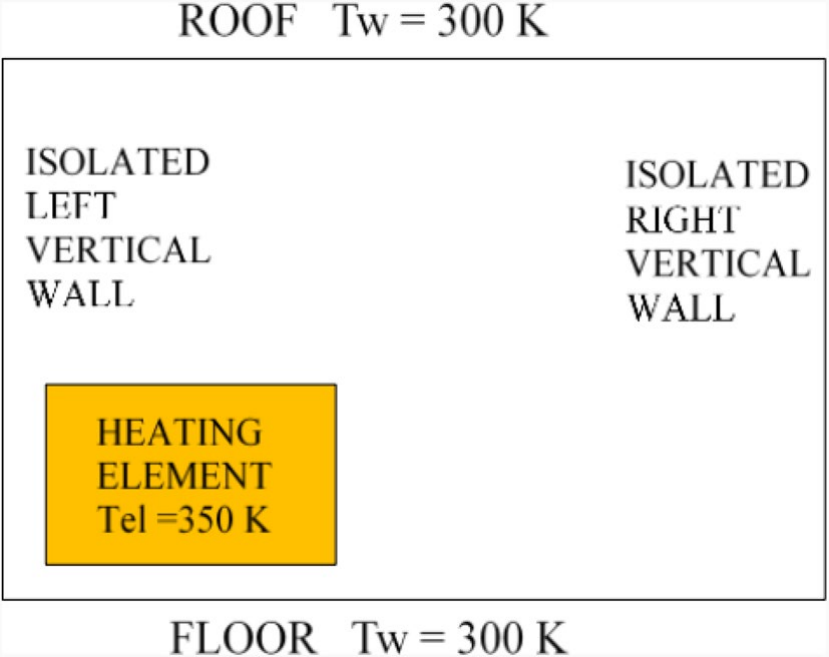
The studied configuration^51^.

To ensure validation and verification, a study was conducted based on the configurations analyzed by Toulouse et al.^51^. Figure 4 illustrates natural convection within a rectangular cavity measuring 0.425 m in height and 0.625 m in length. A rectangular heating element, with dimensions of 0.216 × 0.140 m^2^, is positioned as depicted in the figure. The vertical walls are thermally insulated, while the top and bottom walls are maintained at a constant temperature of 300 K. A thermal distribution of 350 K is applied to the heating rod’s walls, creating a temperature gradient along the cavity’s height.

**Fig. 5:**
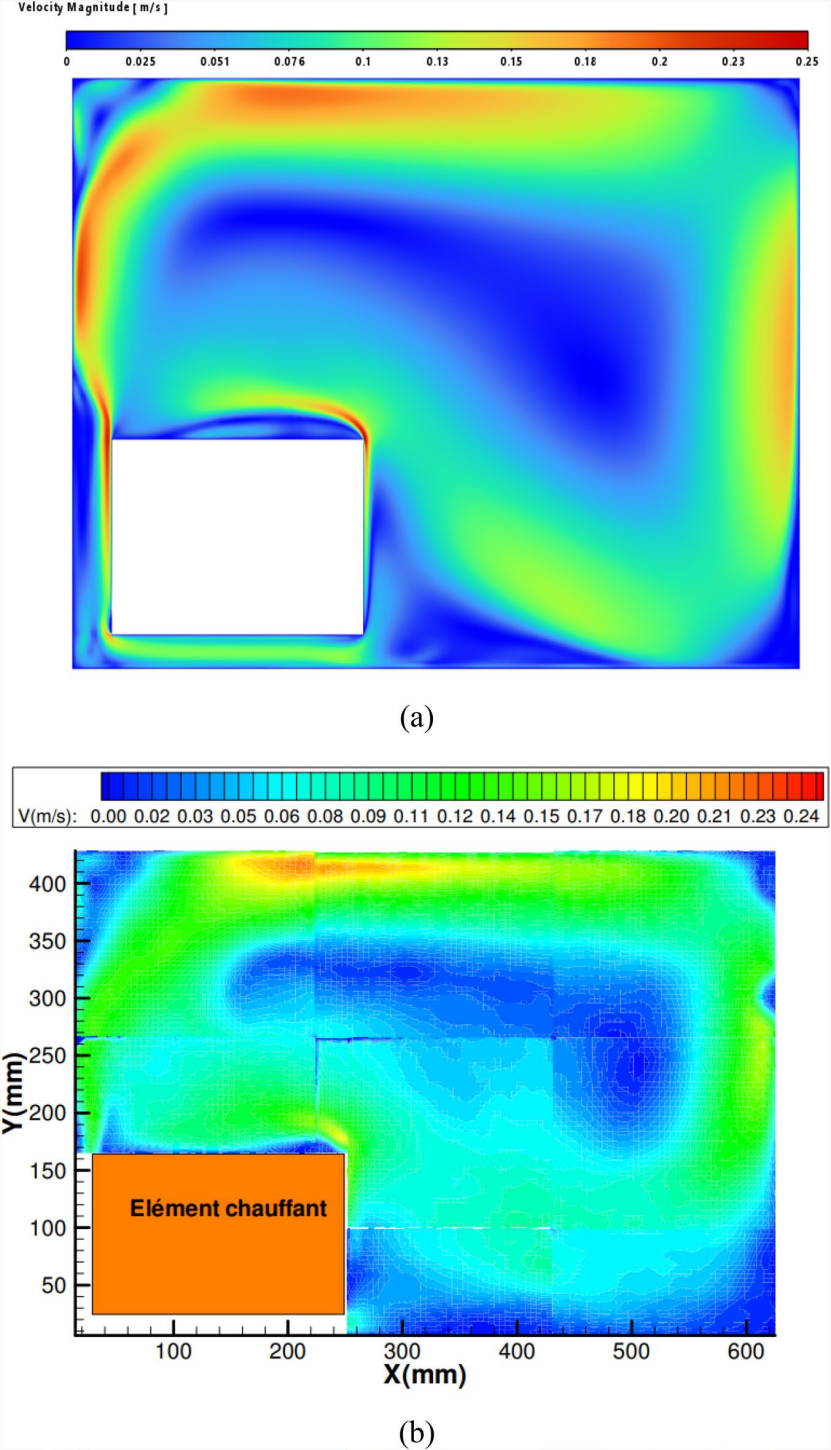
Dynamic field obtained by numerical simulation: (**a**) our numerical results (**b**) experimental results (Toulouse, 2004)^51^.

The flow exhibits a single-cell circulation pattern, with the thermal plume deflecting and detaching from the walls at approximately the same locations within the cavity. Quantitatively, the velocity magnitude is consistent, reaching approximately 0.25 m/s, as shown in Figure 5. Air heats up upon contact with the vertical heated walls, which are maintained at 350 K causing an increase in velocity near the upper corners of the heat source. Numerical simulations performed using the commercial software “Fluent” effectively capture the dynamic behavior of turbulent natural convection in a closed cavity containing a heating rod. A strong agreement is observed between the numerical results obtained in this study and the experimental findings.

**Fig. 6:**
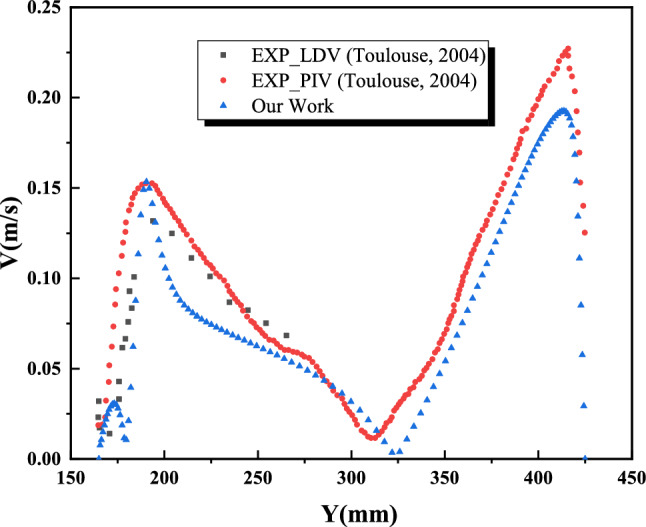
Average velocity profile at X=200 mm.

**Fig. 7:**
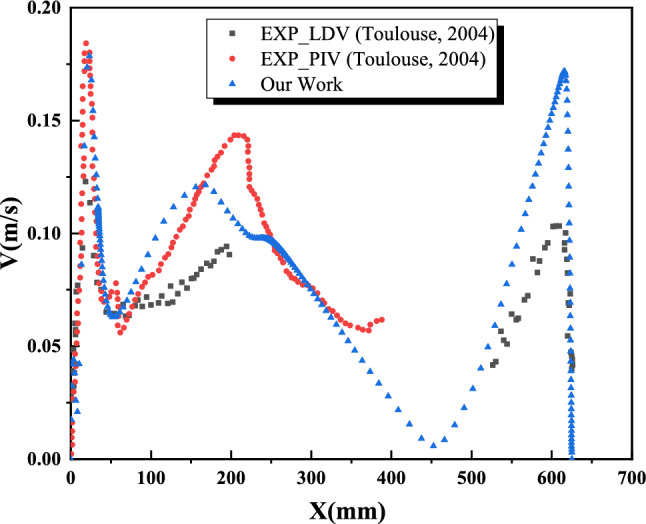
Average velocity profile at Y=200 mm.

Figures 6 and 7 present the numerical and experimental mean velocity profiles at cavity heights of X = 200 mm and Y = 200 mm. Overall, both profiles exhibit similar trends, with a slight underestimation in the numerical results—approximately 9% for X and 10% for Y. This discrepancy can be attributed to the absence of radiation effects in the numerical model.

**Fig. 8:**
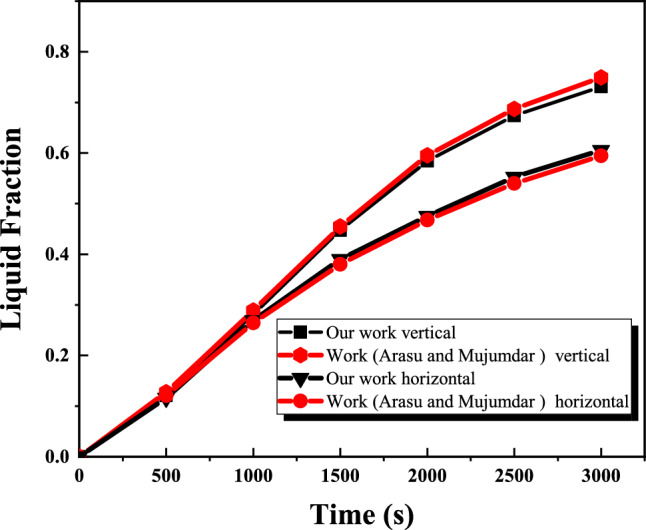
Comparison of numerical model of liquid fraction.

**Fig. 9:**
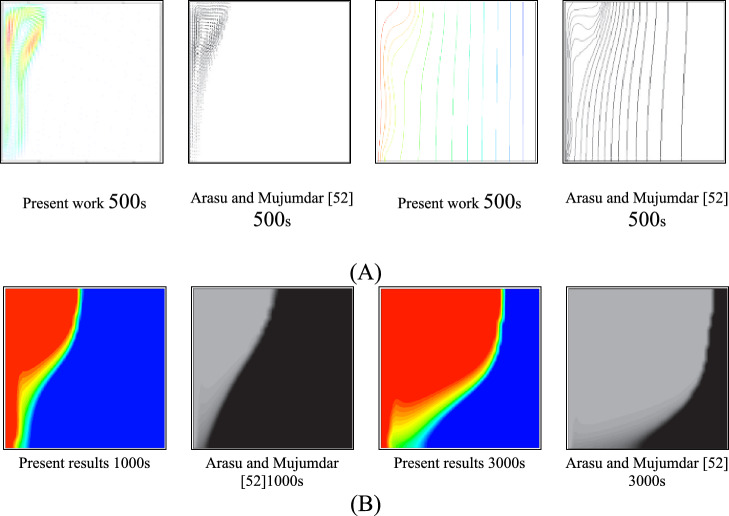
Comparison of results (**A**) in terms of velocity vectors (left) and isotherms (right) (**B**) Liquid-solid interface of paraffin wax.

To validate the computational fluid dynamics (CFD) model applied to the PCM wall, a numerical study was conducted to investigate the melting properties of paraffin wax, a phase change material (PCM) enhanced with 5 wt% aluminum oxide nanoparticles, inside a square container. The simulation results were carefully compared with the data published by Arasu and Mojumdar^52^. Figure 8 highlights the alignment between the fluid fraction calculated by the present model and that reported in the study by Arasu and Mujumdar^52^. As shown in Figure 8, the results show strong consistency with the data from reference^52^. In addition, Figures 9 (A and B) provide a comparative analysis of the velocity vectors and isotherms, comparing the present results with those in the work of Arasu and Mujumdar. Furthermore, the solid-liquid interface at t=1000 s and t=3000 s was compared for paraffin wax. This comprehensive assessment revealed a remarkable level of agreement between the two studies, with discrepancies remaining within a narrow margin of error of 4%.

## Time step and mesh sensitivity study

**Fig. 10:**
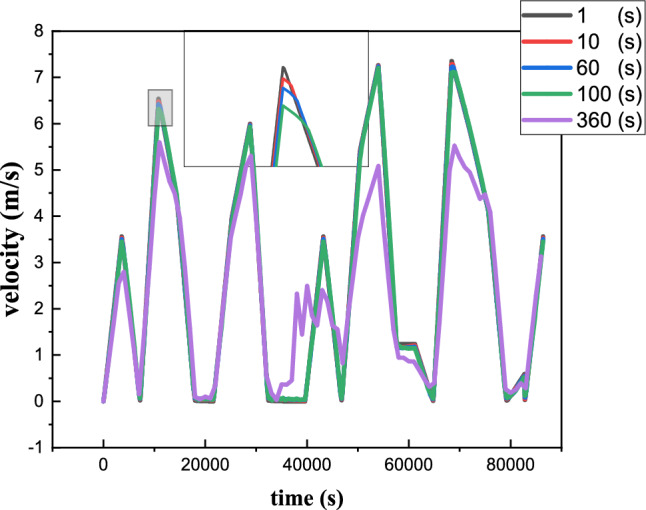
Study of the time step effect.

The numerical solution was systematically studied using five distinct time steps: 1, 10, 100, 180 and 360 seconds. Air outlet velocity was monitored as a function of time for all time steps considered. The results, shown in the figure, reveal negligible differences between time steps of 1, 10 and 60 seconds. However, a clear divergence in the results is observed with the time step of 100 s Figure 10,, and a substantial divergence is evident with the 180-second time step. This observation suggests that the results obtained are consistent for all time steps and independent of network size. Nonetheless, finer time steps enabled greater accuracy to be achieved. As a result, the 10-second solutions were deemed optimal, offering a balance between computer resource efficiency, computation time reduction and error minimization. This selection ensures a satisfactory compromise, enabling accurate simulations while optimizing computing resources.

**Fig. 11:**
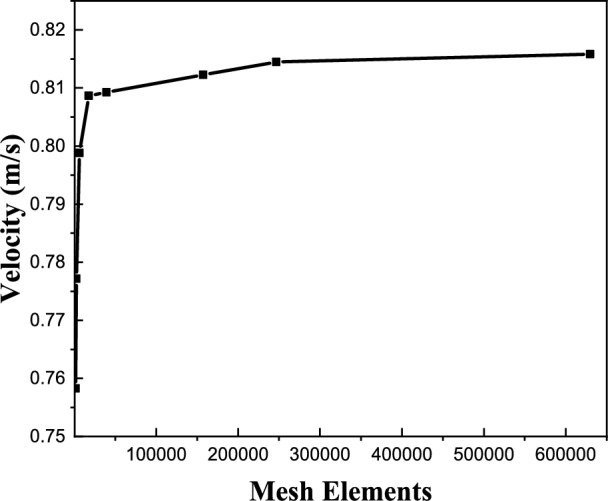
Mesh sensitivity study.

**Fig. 12:**
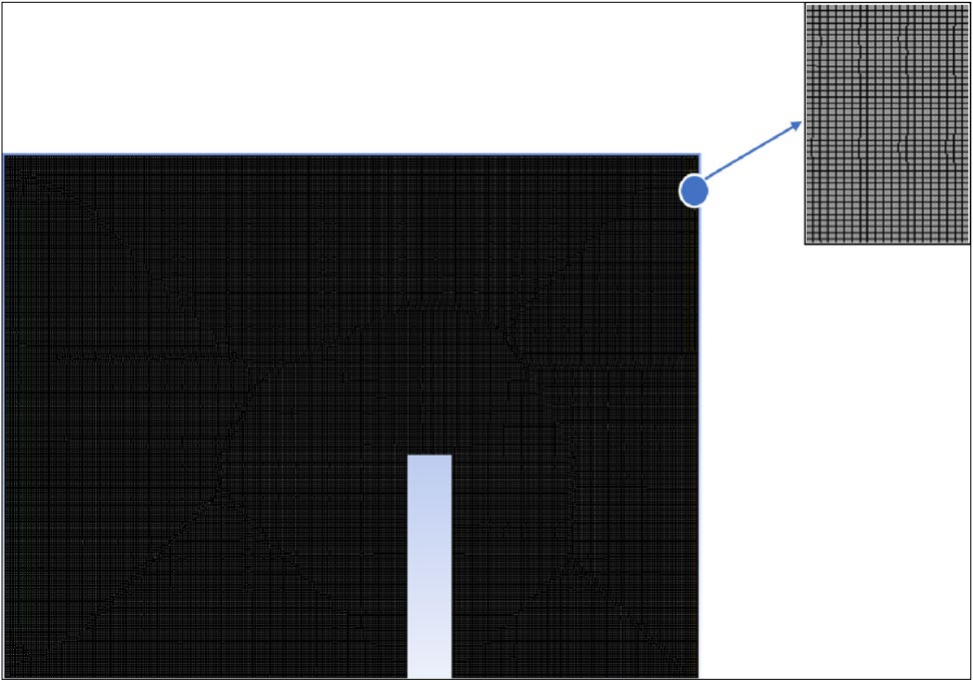
Mesh used in the study.

To ensure numerical accuracy and grid independence, a mesh sensitivity analysis was conducted. The computational domain is two-dimensional, and the mesh consists of unstructured quadrilateral elements with local refinement near the PCM layer, wall interfaces, and ventilation openings to properly resolve velocity and temperature gradients. Eight progressively refined grids were tested, with element numbers of 1575; 2464; 6300; 17550; 39375; 157498; 246564; and 629981. Grid independence was evaluated by monitoring the outlet air velocity under identical boundary conditions. Figures 11 and 12 illustrate the convergence behavior and a close-up of the selected mesh configuration. As the mesh density increased, the variation in outlet velocity decreased significantly. Beyond 246564 elements, further refinement resulted in negligible changes in the monitored parameter, indicating grid-independent behavior. Therefore, the mesh containing 246,564 elements was selected for subsequent simulations, providing an appropriate balance between computational cost and numerical accuracy.

## Results and discussion

The Results and Discussion section clarifies the evaluation framework applied in the Results section. The scope of the comparative analysis is summarised below.

- The most appropriate PCM will be evaluated through a comparative assessment of its thermophysical properties, phase-change behaviour and the resulting thermal comfort indicators, all under identical summer boundary conditions.
- A comparative analysis of the three proposed ventilation configurations was conducted to determine their impact on indoor air temperature distribution, airflow patterns and thermal comfort performance.
- Investigate the effects of wind direction on the selected configuration to identify the most favourable airflow orientation for the climatic conditions in Oum El Bouaghi..

### PCM section

The PCM section focuses on identifying the most suitable material for summer thermal regulation in Oum El Bouaghi’s climate. A comparative parametric evaluation was conducted for the selected PCMs, considering interior surface temperature response, phase-change behaviour, liquid fraction evolution and daily integrated heat flux reduction. The aim is not to carry out a formal optimisation process, but rather to determine which PCM performs best among the investigated candidates under identical boundary conditions, taking into account both thermal stability and indicative material cost.

**Fig. 13:**
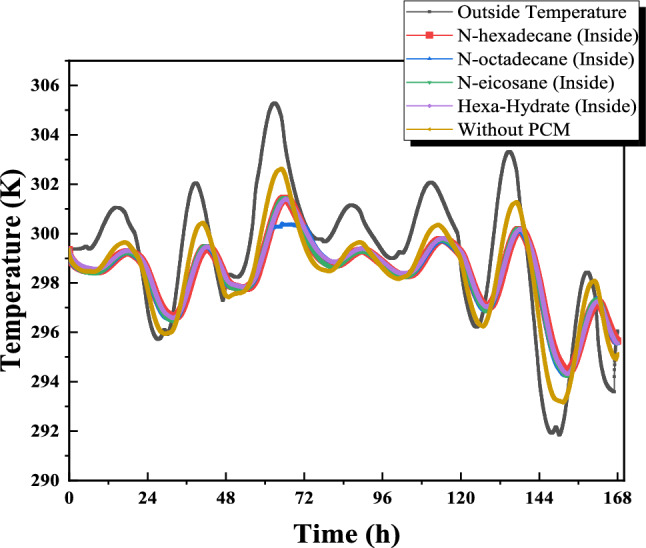
Interior and exterior wall temperature distribution over time (days in July).

Figure 13 presents the measured exterior wall surface temperatures and ambient air temperatures for July, which were used as boundary conditions for the CFD simulations. In the numerical model, the measured outdoor temperature was imposed on the exterior (left) side of the wall, while the interior (right) side was analyzed to evaluate thermal response. The investigated wall configuration consisted of brick combined with a 2.5 cm PCM layer. Three PCMs—n-octadecane, n-eicosane, and n-hexadecane—were examined, and their thermophysical properties are summarized in Table 1. The July measurements reveal a typical diurnal pattern, with higher exterior surface temperatures during daytime and reversed gradients during nighttime. All PCM-enhanced walls reduced indoor temperature fluctuations compared to the reference wall without PCM, confirming the beneficial latent heat effect. Among the tested materials, n-hexadecane produced the lowest interior surface temperatures during peak solar loading due to its lower melting temperature and rapid phase transition. However, n-octadecane exhibited more stable thermal behavior over the full daily cycle, maintaining temperatures closer to the comfort range for a longer duration. This improved temporal stability highlights its suitability for summer thermal regulation under the studied climatic conditions..

**Fig. 14:**
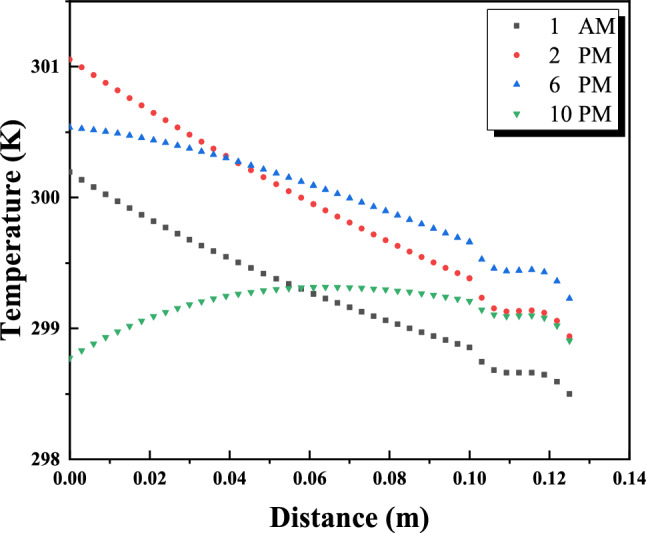
Temperature distribution along the wall for different hours of the day.

Figure 14 shows the transient temperature changes crossing a wall consisting of brick and a layer of phase change material (PCM), specifically n-hexadecane. Heat from the left side of the wall warms the outside of the wall, and this thermal energy is transferred to the interior through conduction, gradually raising the interior temperature. Conversely, a decrease in temperature at night causes heat to be transferred to the outside, which leads to a decrease in the internal temperature. Inserting a layer of PCM next to the brick changes the temperature profile of the wall, as shown in Figure 14. The application of external heat is intended to liquefy the PCM upon arrival, maintaining its melting point while the PCM remains solid. This layer acts as a thermal barrier, blocking the flow of heat from the outside of the wall. Properly measuring the thickness of the PCM layer - ensuring partial melting until sunset - ensures that the internal temperature does not fluctuate due to external heat transfer. During night cooling, the external wall temperature decreases, allowing the heat of the molten PCM to be dissipated to the external environment, maintaining internal ambient conditions. Notably, Figure 14 reveals a positive effect on the 0.5 cm thick inner layer of PCM, but it is not suitable for complete insulation, which necessitates increasing the thickness of the PCM layer.

**Fig. 15:**
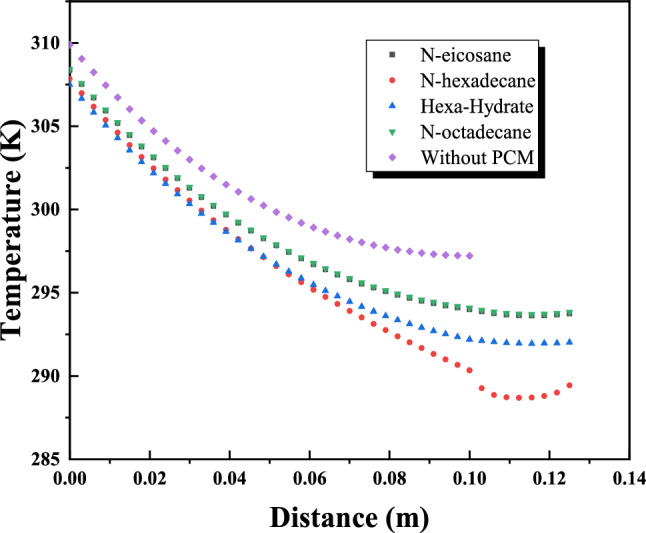
Temperature distribution along the wall for PCMs studied.

Figure 15 presents the transient temperature distribution across the investigated wall configuration, consisting of a brick layer combined with a 2.5 cm internal PCM layer. The objective of this analysis is to compare the thermal response of different PCMs under representative July conditions, rather than to perform a formal optimization. A representative peak hour was selected to examine the temperature gradient through the wall and to compare PCM-enhanced cases with the reference wall (10 cm brick without PCM). As previously described, the outdoor air temperature applied on the exterior side (left boundary) increases the outer wall surface temperature, and heat is transferred inward by conduction. In the reference case, this leads to a higher interior surface temperature due to the absence of latent heat buffering. The integration of PCMs modifies this behavior by introducing latent heat absorption during phase transition, which reduces the temperature gradient across the wall. Among the investigated materials, n-hexadecane produced the lowest instantaneous interior surface temperature at the selected hour, reflecting its rapid phase transition and strong short-term heat absorption capacity. In contrast, n-octadecane maintained an interior temperature closer to the comfort reference temperature (297 K), indicating a more moderated and stable thermal response. This comparison highlights the difference between peak temperature reduction (more pronounced for n-hexadecane) and sustained thermal regulation (more evident for n-octadecane), which is critical when evaluating PCM suitability for hot arid summer conditions.

**Fig. 16:**
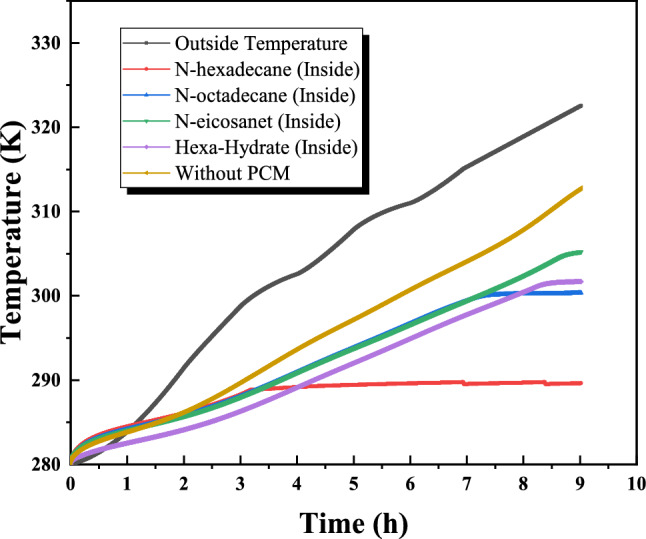
Distribution of interior and exterior wall temperature over time.

Figure 16 illustrates the temporal evolution of interior and exterior wall surface temperatures under representative July solar conditions. The exterior surface was subjected to measured outdoor temperature and solar exposure typical of the study area, enabling comparison between the reference wall (without PCM) and PCM-enhanced configurations. In the reference case, the increase in exterior surface temperature during peak solar hours resulted in a corresponding rise in the interior surface temperature, although attenuated by the thermal resistance of the brick layer. In contrast, PCM-integrated walls exhibited moderated interior temperature growth due to latent heat absorption during the phase-change process. among the investigated materials, n-hexadecane demonstrated the strongest instantaneous temperature stabilization effect, reaching a quasi-isothermal plateau near its melting temperature after approximately 3.5 hours of exposure. This behavior reflects rapid latent heat absorption during peak heating. However, due to its relatively lower melting temperature, the phase-change process was completed earlier in the day. Conversely, n-octadecane exhibited a more gradual temperature evolution and maintained phase-change activity over a longer duration, resulting in improved thermal stability throughout the daily cycle. This extended stabilization period contributes to reducing indoor temperature fluctuations during peak hours, overall, PCM integration reduced overheating compared to the reference wall, confirming the role of latent heat storage in passive thermal regulation. While the present study does not explicitly model HVAC energy consumption, the observed reduction in peak interior surface temperatures suggests a potential decrease in cooling demand under similar climatic conditions. Further investigation is required to quantify long-term seasonal performance and full energy savings.

**Fig. 17:**
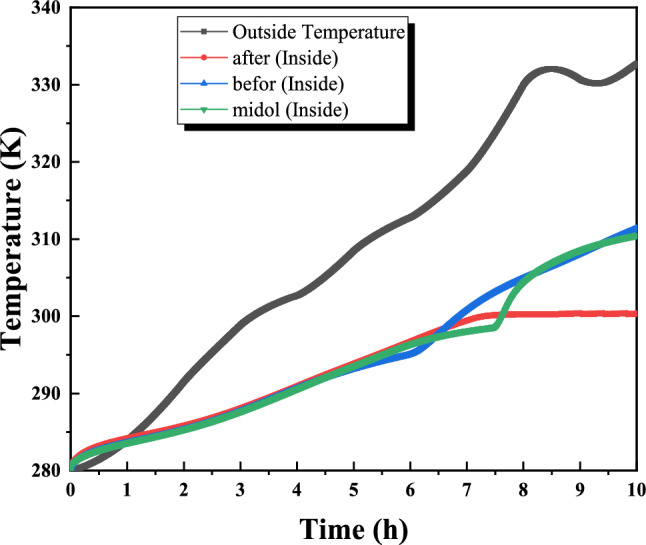
Interior and exterior wall temperature distribution over time (comparing the position of the PCM layer in the wall) (solar radiation application during summer day).

Figure 17 displays the inner and outer wall surface temperatures of a specific brick wall combined with a phase change material (PCM) layer. The experiment involved applying the PCM layer at different locations on the brick wall: once on the inner side, once on the outer side, and once in the middle. Next, sunlight exposure was directed toward the exterior wall for several hours to evaluate the most effective PCM layer placement. The results reveal noticeable patterns: rapid increases in the external temperature, and all the solutions studied work to reduce the internal temperature. However, when the PCM layer is located externally, the internal temperature quickly exceeds the melting point of the PCM, causing the internal temperature to rise. On the contrary, the two alternative solutions, especially when the PCM layer is on the inner side, take advantage of the low conductivity of the bricks, delaying the inner temperature from exceeding the melting threshold. Thus, the wall equipped with an inner PCM layer keeps the internal temperature constant at the melting point.

**Fig. 18:**
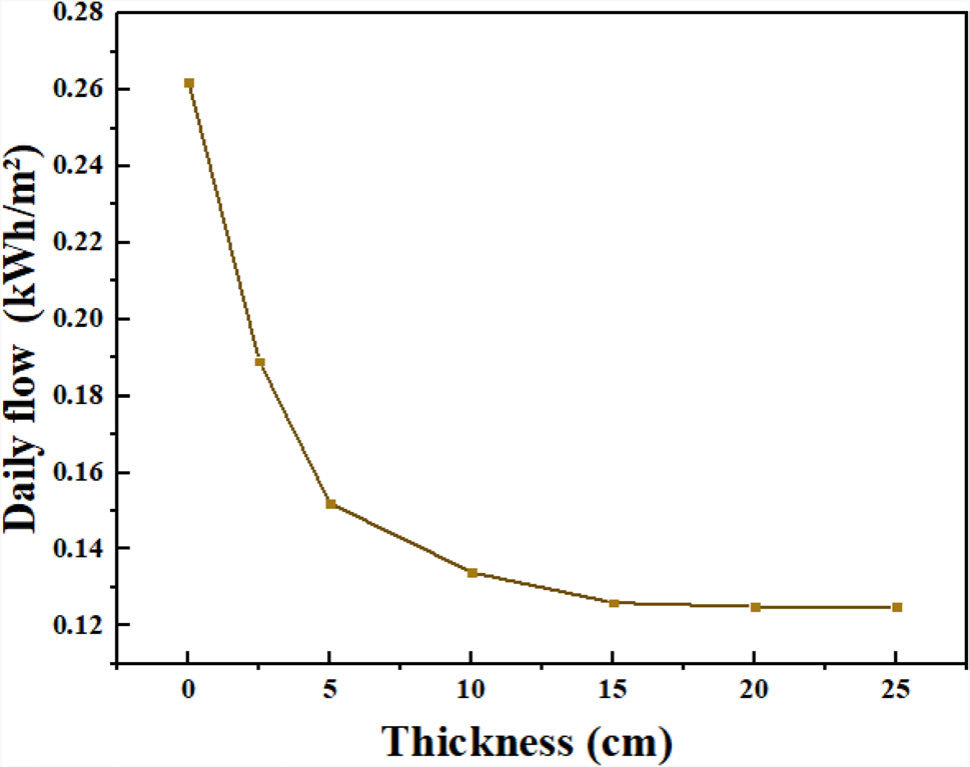
Effect of PCM thickness on daily integrated heat flux through the wall.

Figure 18 presents the daily integrated heat flux transmitted through the brick wall incorporating an internal n-hexadecane layer for different PCM thicknesses, while maintaining constant brick thickness and PCM placement. Increasing PCM thickness results in a progressive reduction in daily heat flux transmitted to the interior. Compared to the brick-only reference wall (0 cm PCM), the reductions reach 28.0% for 2.5 cm, 42.0% for 5 cm, 49.0% for 10 cm, and 52.0% for 15 cm under July cooling-dominant boundary conditions. However, beyond 10–15 cm, the incremental improvement becomes marginal, indicating diminishing thermal returns. This behavior can be attributed to the limited depth of daily thermal penetration, whereby only a portion of the PCM layer actively undergoes phase change during the diurnal cycle. Consequently, the 10–15 cm range represents the upper bound of thermal benefit within the present parametric investigation. From an engineering feasibility perspective, thinner PCM layers (e.g., 5–10 cm) may offer a more practical compromise between constructability, structural integration, and thermal performance.

**Fig. 19:**
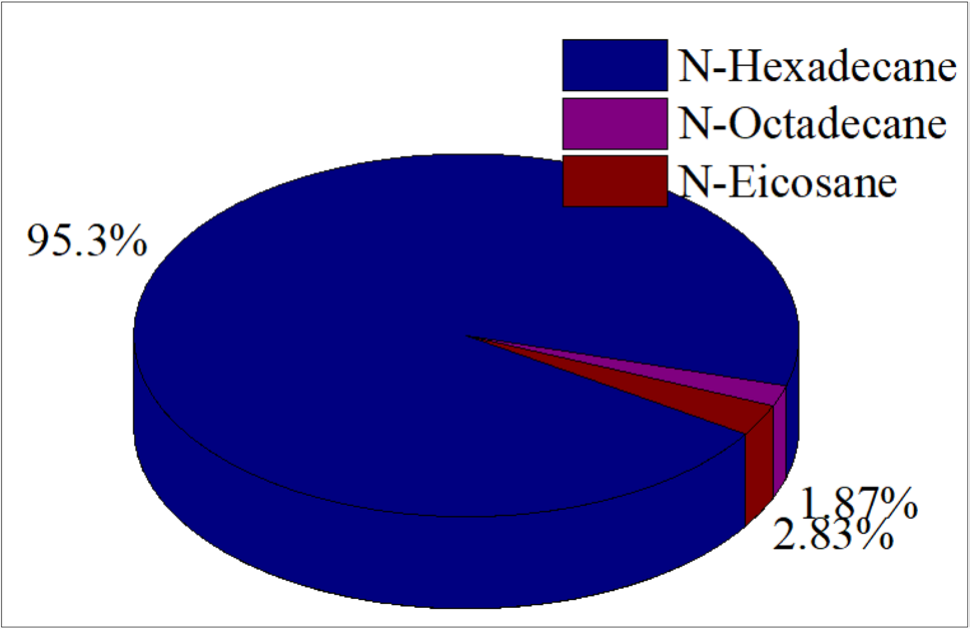
Cost comparison of n-Hexadecane, n-Octadecane, and n-Eicosane per Gram.

The economic feasibility of phase change materials (PCMs) in building applications has been widely discussed in the literature. Jeeja Jacob et al.^53^ reported that PVT–PCM systems can be financially viable when acceptable payback periods are achieved. Similarly, S. K. Jha et al.^54^ demonstrated that integrating PCMs into building envelopes can reduce energy-related expenses by decreasing reliance on mechanical heating and cooling systems. Abrar Ahmad et al.^55^ showed that, under specific climatic conditions, payback periods may approach five years, particularly when PCM systems are combined with controlled natural ventilation strategies. Furthermore, Lianying Zhang et al.^56^ indicated that PCM-integrated exterior walls without excessive additional insulation may provide improved economic and environmental performance compared to heavily insulated PCM configurations. In the present study, a full techno-economic or lifecycle cost analysis was not conducted. Instead, an indicative comparison of unit material prices per kilogram was performed based on supplier data at comparable purity levels^57^. According to these data, n-hexadecane exhibits the highest unit material cost, while n-octadecane and n-eicosane are substantially less expensive on a per-kilogram basis Figure 19. This comparison reflects raw material pricing only and does not account for encapsulation, installation, maintenance, or lifecycle costs. Nevertheless, when considered alongside the thermal performance results, the observed material-level cost differences highlight the importance of evaluating both thermophysical behavior and indicative material affordability when selecting a PCM for summer-dominant building applications in climates such as Oum El Bouaghi.

**Fig. 20:**
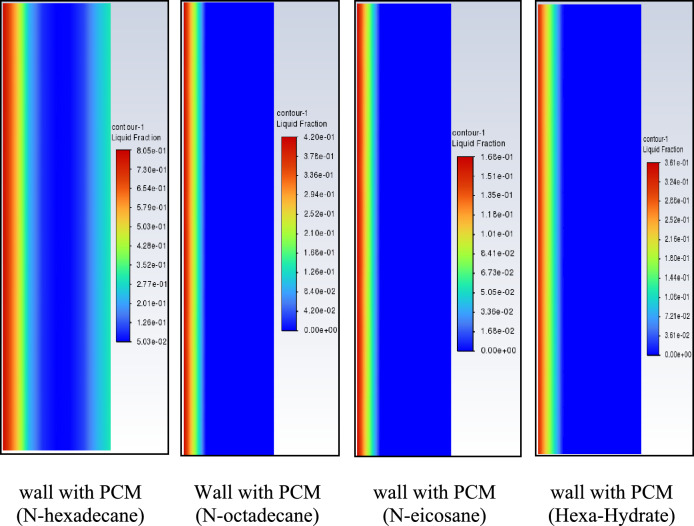
The contents of liquid fraction.

Figure 20 presents the liquid fraction distribution within each PCM layer at midday in July. The liquid fraction represents the proportion of material that has undergone phase transition at the selected time. Among the investigated materials, n-hexadecane exhibits the highest liquid fraction (approximately 0.8), indicating rapid and extensive phase change during peak solar exposure. This behavior confirms its strong instantaneous latent heat absorption capacity. Hexahydrate and n-eicosane show moderate phase transition levels, while n-octadecane displays a more gradual melting process at the same time step. The higher liquid fraction of n-hexadecane explains its ability to produce lower interior surface temperatures during peak hours. However, this rapid melting also implies earlier completion of the phase-change process, which may limit its thermal buffering capacity later in the day. In contrast, n-octadecane undergoes a more progressive phase transition, contributing to extended thermal regulation over the daily cycle.

**Fig. 21:**
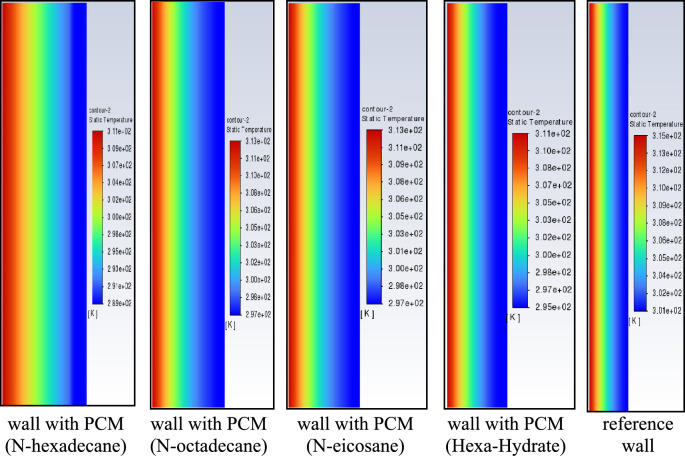
Contours of the isotherms after 6 hours.

To further evaluate the thermal response of walls incorporating n-octadecane, n-eicosane, and n-hexadecane, temperature distributions across the wall thickness were analyzed under external heat exposure. Figure 21 illustrates the two–dimensional temperature fields at t=6 hours for the reference brick wall and PCM-enhanced configurations. In the reference case (brick only), heat absorbed at the exterior surface propagates inward by conduction, resulting in an interior temperature of approximately 301 K after 6 hours. The thermal inertia of the brick moderates but does not prevent heat transfer toward the interior. When a PCM layer is integrated on the inner face of the wall, latent heat absorption modifies the temperature distribution. The addition of PCM reduces the interior surface temperature relative to the reference case, with temperature differences ranging approximately from 4 K to 12 K depending on the material. The largest instantaneous temperature reduction is observed for n-hexadecane, consistent with its high liquid fraction and rapid phase transition. However, n-octadecane provides a more uniform temperature gradient across the wall thickness, reflecting a more stable thermal buffering effect. These results reinforce the distinction between peak temperature attenuation more pronounced for n-hexadecane and sustained daily thermal stability more evident for n-octadecane under the investigated summer conditions.

### Ventilation section

**Fig. 22:**
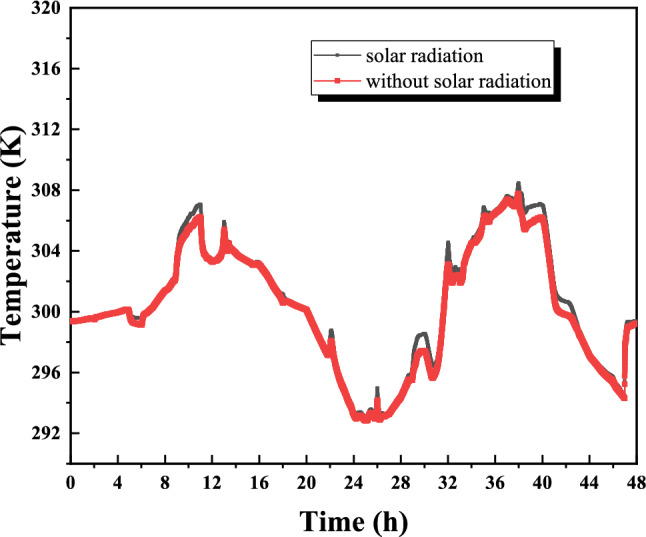
Comparison of indoor air temperature evolution with and without solar radiation under identical boundary conditions for sensitivity assessment.

In this study, we investigated the performance of various building ventilation configurations under distinct weather conditions in Oum El Bouaghi, Algeria. To streamline the analysis and optimize computational efficiency, solar radiation was excluded from the weather conditions, based on preliminary findings indicating its negligible impact on the specific ventilation configurations examined. Climate-adaptive ventilation refers to adjusting the position and operation of vents according to prevailing wind direction and time of day, thereby enhancing heat removal when outdoor air offers cooling potential, Climate-adaptive ventilation refers to adjusting the position and operation of inlet and outlet vents according to prevailing wind direction and daily cooling capacity. Figure 22 illustrates the comparative effects of solar radiation on the temperature dynamics within the studied room, delineating the temperature variations with and without the influence of solar radiation. The results encompass a comprehensive dataset, including average temperature measurements across different configurations and specific zones, detailed temperature profiles, air velocity distributions, and Effective Draft Temperature (EDT) parameters. Additionally, the study evaluates the synergistic interactions between these variables to provide a holistic understanding of the ventilation efficacy.

To justify the exclusion of solar radiation in the comparative ventilation analysis, a preliminary sensitivity assessment was conducted. Figure 22 compares the indoor thermal response with and without solar radiation under identical boundary conditions. The results indicate that including solar radiation leads to a variation of less than 3% in average indoor air temperature and does not alter the ranking of ventilation configurations in terms of temperature distribution, air velocity patterns, or Effective Draft Temperature (EDT). Since the objective of the ventilation section is to compare the relative performance of airflow configurations under identical external thermal conditions, and solar radiation affects all configurations similarly, it was excluded from the main ventilation simulations to reduce computational cost while preserving.

#### Configuration tests

The configuration tests section evaluates the thermal and airflow performance of the proposed ventilation layouts under identical boundary conditions. The aim is to compare the influence of different inlet–outlet arrangements on indoor temperature distribution, air velocity patterns, Effective Draft Temperature (EDT) and synergy behaviour. Rather than being an optimisation procedure, this analysis is conducted as a comparative assessment, with the aim of identifying the configuration that provides the most stable and thermally comfortable indoor environment under the prevailing summer conditions in Oum El Bouaghi.

**Fig. 23:**
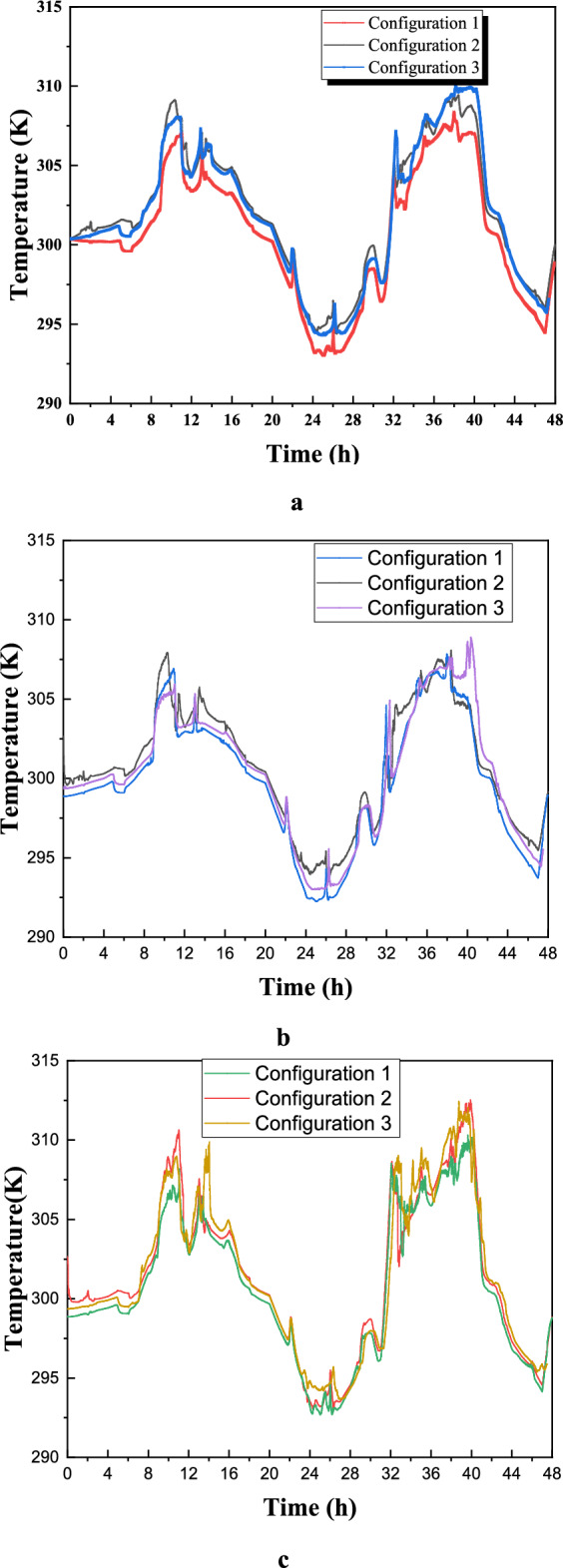
Distribution of air temperature inside the room over time (days in July). (**a**): Average temperature in the room; (**b**): Position (**c**); C: Position B.

Figure 23(a) illustrates the variation in average air temperature over 48 hours across different selected configurations. Configuration 1 consistently maintains lower temperature values compared to the others, indicating superior thermal management in mitigating temperature peaks. In contrast, Configuration 3 exhibits the highest temperatures, reflecting reduced effectiveness in maintaining thermal stability. Between hours 8 and 24, the temperature curves highlight the influence of external conditions and thermal inertia, with a noticeable drop around hour 20, likely due to nocturnal cooling. Temperature peaks around hours 12 and 36 suggest diurnal heating caused by solar gains or internal heat generation. The smoother temperature profile in Configuration 1 suggests enhanced thermal regulation, potentially attributed to optimized ventilation or favorable material properties, whereas the pronounced fluctuations in Configuration 3 indicate greater sensitivity to heat flux variations. Configuration 2 demonstrates an intermediate performance, balancing the thermal effects observed in the other two cases. To improve the accuracy of assessing occupant comfort, temperature measurements were taken at two distinct points within the room—one near the air inlet (point A) and another farther away (point B). Figures 2223(b) and (c) confirm the previous findings, demonstrating Configuration 1’s effectiveness in maintaining thermal comfort. Notably, the temperature difference between points A and B reached approximately 3 K, with point B experiencing higher temperatures due to its distance from the air inlet. These results underscore the significance of balanced airflow distribution in preventing localized discomfort, particularly in workspaces where thermal uniformity impacts productivity. Furthermore, Figure 23 highlights Configuration 1’s practical advantages for building performance and occupant comfort, as its ability to mitigate temperature peaks suggests optimized use of thermal mass, ventilation, or passive cooling strategies. This could substantially reduce energy consumption and peak HVAC loads compared to the heat-sensitive design of Configuration 3. Additionally, Configuration 1’s stable diurnal profile suggests potential for night purging in climates with cool evenings, further enhancing energy efficiency. These findings advocate for climate-adaptive building designs that integrate high thermal inertia materials and strategic ventilation while emphasizing the need for retrofitting less effective structures—such as Configuration 3—through insulation or shading enhancements. However, real-world implementation must account for cost-benefit trade-offs and occupant behavior to maximize energy savings.

**Fig. 24:**
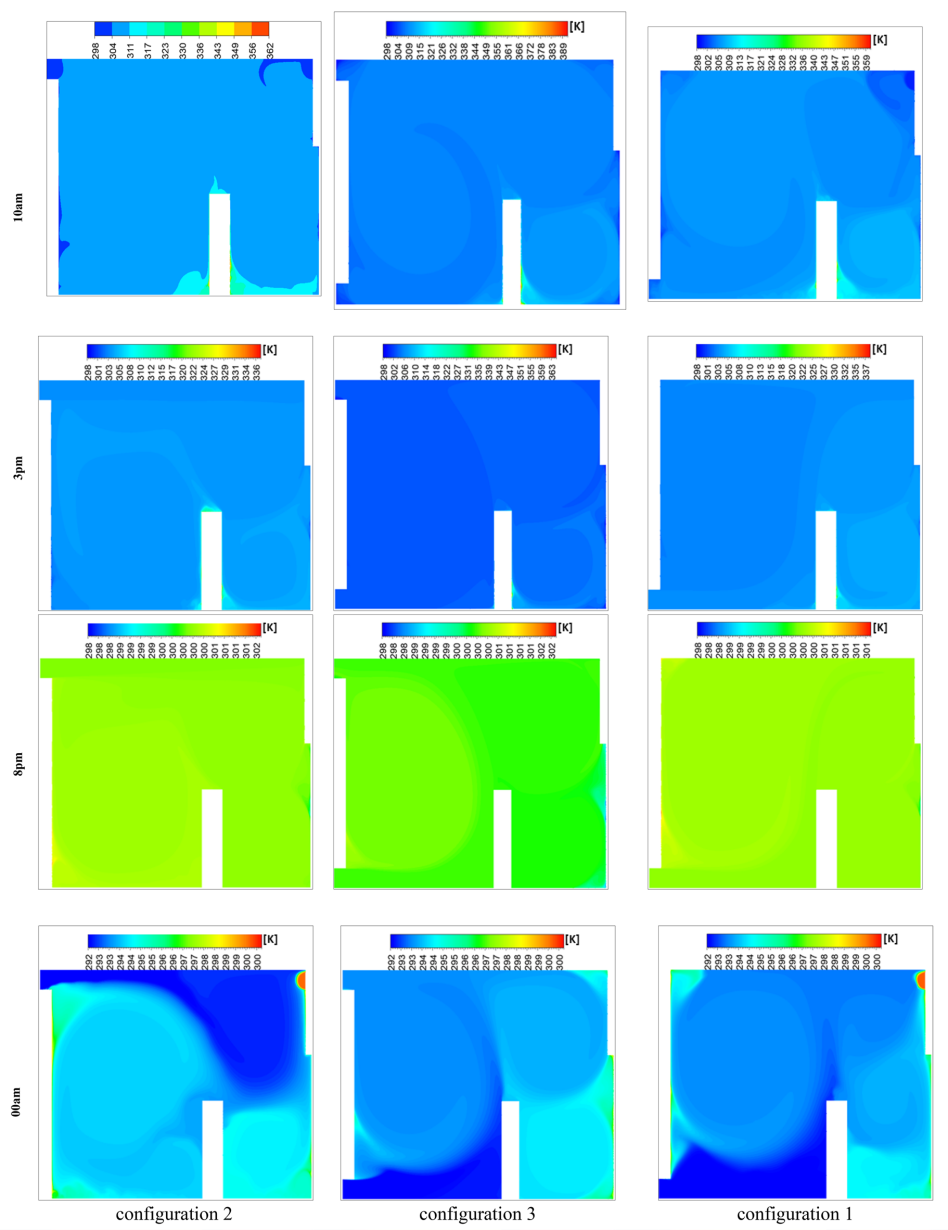
Contours of the isotherms at different hours for all configurations.

Figure 24 presents the isotherm profiles for all the studied configurations at different hours of the day, offering critical insights into temperature distributions. The display of these features throughout the day serves as a pivotal indicator for evaluating the effectiveness of the studied configurations. Notably, the configuration at 10 a.m. maintains a consistent room temperature of 311 K, extending throughout the entire room, with outlet values at 300 K. In contrast, configuration 3 records the same room temperature but exhibits higher values on the right side of the room. Specifically, configuration 2 showcases temperature values ranging between 312 and 319 K during the same hour. At 15 o’clock, both the first and third configurations exhibit elevated values on the right side of the room compared to the rest of the space. Both configurations 1 and 2 have values close to 301 K for the entire room at 20 o’clock. In contrast, configuration 3 at 15 o’clock displays values of 301 K in the wind trajectory inside the room and values close to 311 K in the remaining areas. By 24 o’clock, configuration 2 records values around 292 K in the wind trajectory and the upper right side, while the rest of the room exhibits values close to 296 K. Moreover, configurations 1 and 3 document values of 292 K on the lower left side of the room and 296 K on the right side,This detailed analysis of isotherm profiles provides crucial insights into the thermal performance of each configuration across different hours of the day. The variations in temperature distribution highlight the potential strengths and limitations of each configuration, guiding decisions on the most effective strategies for maintaining thermal comfort in the studied environment.

**Fig. 25:**
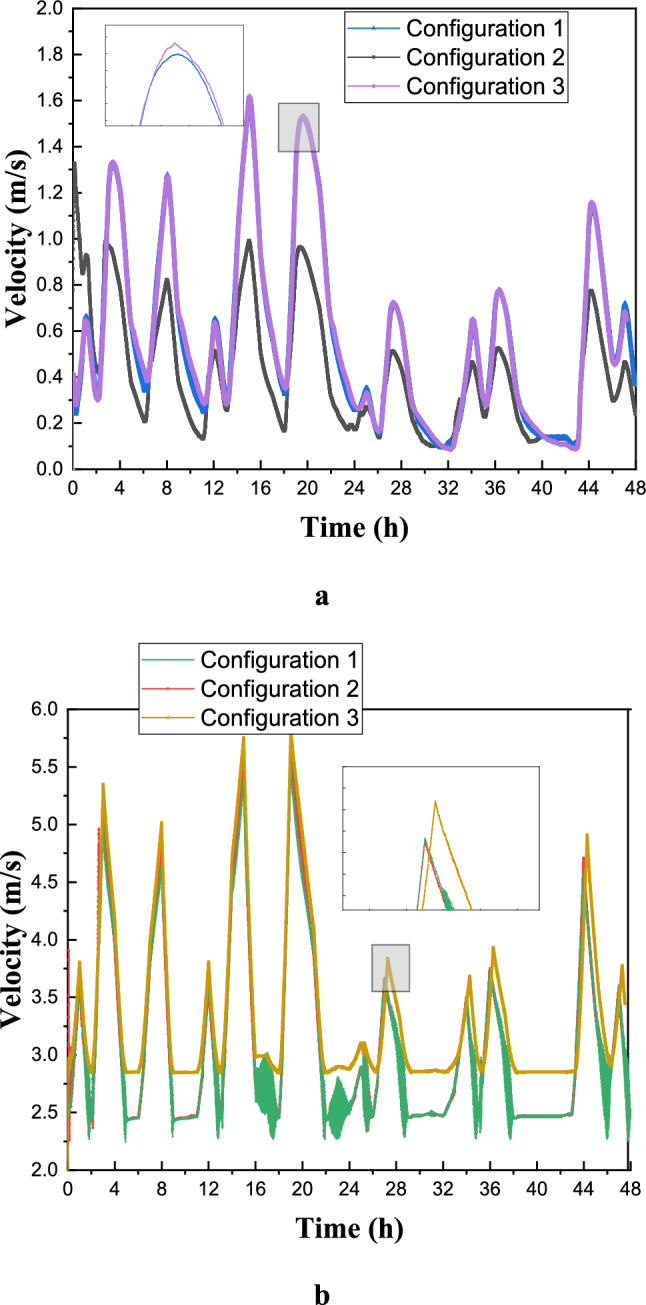
Average air speed for all configurations (**a**): in the room; (**b**): at the outlet.

Figure 25 illustrates the average air speed for all configurations, with Figure 25(a) showing the distribution of air velocities inside the room and Figure 25(b) displaying the air velocity at the outlet. Despite maintaining a constant inlet velocity across all studied cases, significant differences in indoor air velocity arise due to variations in inlet and outlet port placements. Notably, while the directional pattern of airflow and outlet velocity remains relatively stable, temporal analysis reveals distinct airflow behaviors across configurations. Configuration 1 exhibits a stable and consistent velocity profile with minimal fluctuations, indicating a well-balanced and continuous airflow. Configuration 2, on the other hand, demonstrates periodic peaks and troughs, reflecting dynamic adjustments in response to changing conditions. Configuration 3 shows an initial increase in air velocity before stabilizing, highlighting its ability to adapt and gradually reach a steady airflow regime over time. These variations underscore differences in dynamic performance, with Configuration 1 ensuring stability, Configuration 2 responding adaptively to changes, and Configuration 3 balancing adaptation with stability. Figure 2425(b) further reveals that Configuration 3 achieves the highest average air velocity at the outlet, slightly surpassing Configuration 2 and showing a marginal discrepancy from Configuration 1. When the inlet and outlet ports are positioned along the same horizontal plane, the variation in their horizontal positioning or placement along the edges of the same wall influences room temperature distribution, leading to increased average air velocity. This relationship between ventilation design and indoor airflow characteristics has significant practical implications for optimizing indoor environmental quality. The air velocity analysis indicates that Configuration 1’s stable airflow pattern reflects a well-balanced ventilation strategy, making it suitable for environments that require steady thermal comfort and effective contaminant control, such as hospitals or laboratories where minimizing drafts is essential. Configuration 2’s fluctuating airflow, while less stable, offers adaptability for spaces with variable occupancy or thermal loads, making it suitable for demand-responsive ventilation systems in offices or classrooms, potentially improving energy efficiency. Configuration 3’s gradual stabilization at higher outlet velocities indicates strong air mixing, making it ideal for industrial settings or kitchens where efficient pollutant extraction is necessary. Additionally, the positioning of inlets and outlets plays a crucial role in ventilation efficiency, with higher velocities observed in configurations where these elements share a horizontal plane, suggesting improved air exchange rates. However, higher air speeds in Configuration 3 must be carefully managed to prevent discomfort, particularly in sedentary environments. These findings emphasize the necessity of tailoring ventilation strategies to specific spatial requirements—where Configuration 1 supports precision-controlled environments, Configuration 2 serves dynamic spaces with fluctuating conditions, and Configuration 3 ensures effective mixing in contamination-prone areas.

**Fig. 26:**
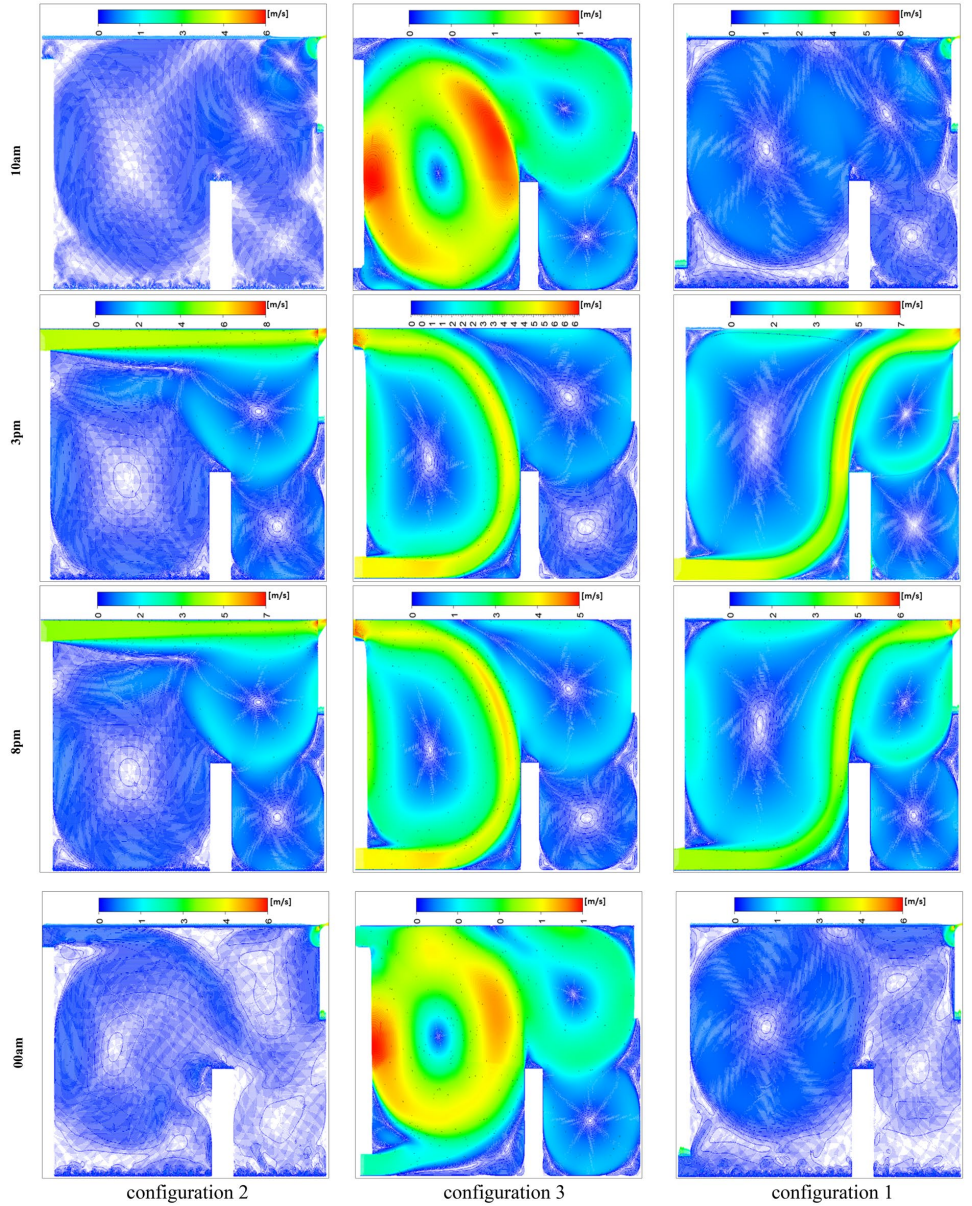
Contours of the velocity at different hours for all configurations.

Figure 26 illustrates the complex distribution of air velocity under the selected configurations, derived from simulations conducted at various time frames during both day and night. The observation planes were selected to highlight the distinctive airflow characteristics that arise when the inlet and outlet configurations are favorably aligned. This alignment generates a powerful unidirectional air stream, free from secondary drafts, which enhances overall air velocity within the room. The unidirectional flow minimizes the mixing of indoor air and reduces heat transfer to the exterior, thereby improving thermal efficiency. Notably, Configuration 1 exhibits a unique pattern of secondary air streams distributed throughout the room, promoting more extensive air movement. This increased circulation facilitates heat expulsion, aligning with and reinforcing earlier findings. In contrast, Configuration 3, which positions the inlet and outlet on the same wall, shows fewer instances of secondary drafts, resulting in limited air movement and reduced heat dissipation. This comparative analysis underscores the significant impact of inlet and outlet configurations on the generation of secondary currents and their role in heat management within the room.

**Fig. 27:**
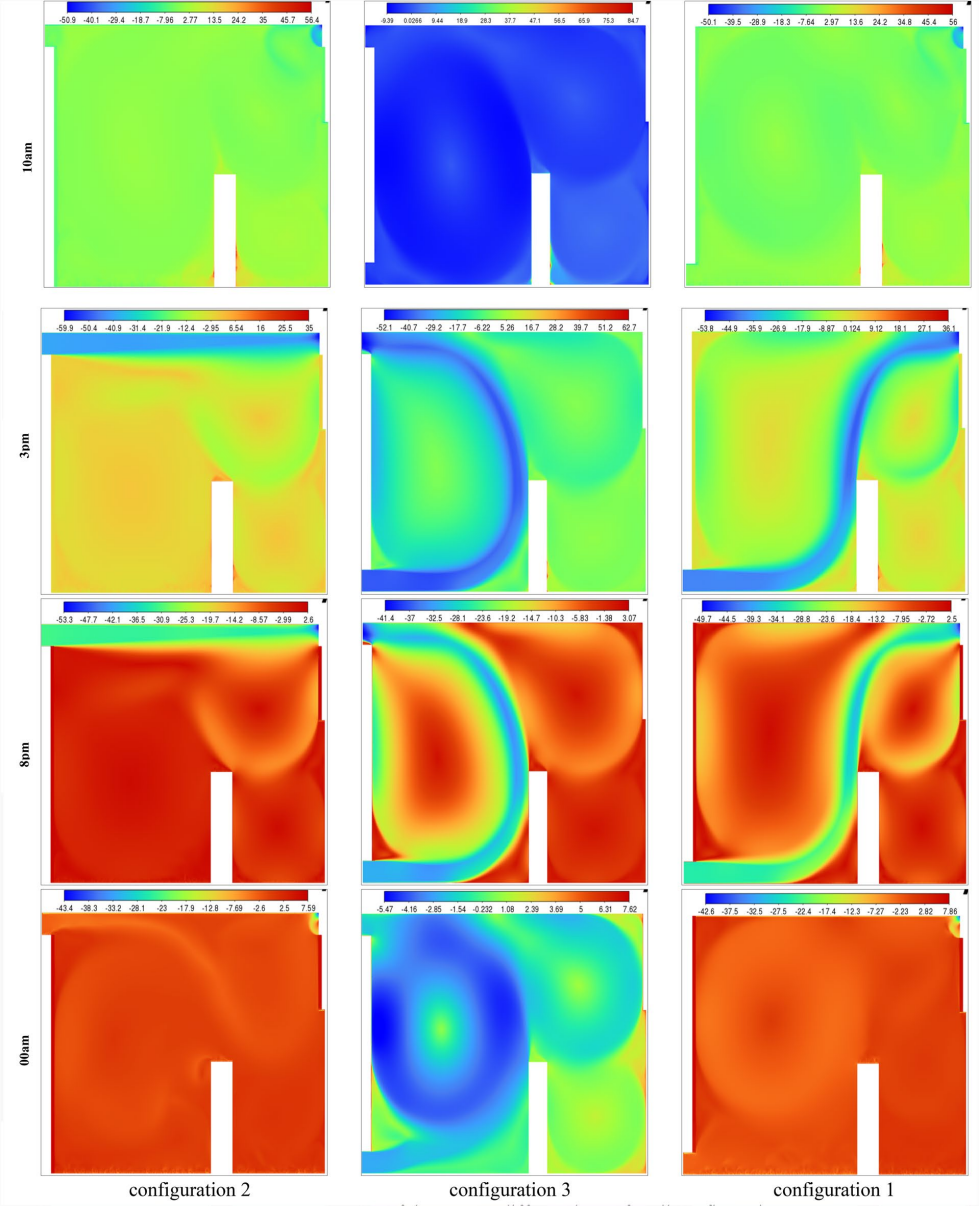
Contours of the EDT at different hours for all configurations.

The Effective Draft Temperature (EDT) index is a critical metric for evaluating thermal comfort zones, as it integrates two key parameters: air temperature and velocity, as defined in Formula (16). The EDT index is highly sensitive to fluctuations in these parameters, particularly when they fall outside the established thermal comfort range. EDT values exceeding +1.1 indicate a transition into an uncomfortably warm zone, while values below −1.7 signify a shift toward a cold discomfort zone. Values within the range of −1.7 to +1.1 are considered to fall within the thermal comfort zone for occupants. Figure 27 depicts the thermal comfort zones across all configurations at various times of the day and night. Configuration 2, which positions the air inlet and outlet on opposite sides of the ceiling, exhibits a pronounced cold discomfort zone near the inlet. This zone extends along the upper portion of the room, likely due to the interplay between buoyancy forces and airflow, which disrupts convective patterns. However, the middle and lower regions of the room remain within the thermal comfort zone, ensuring comfort for centrally seated occupants, despite a slight cold sensation in the upper area. In Configuration 1, where the air inlet is located at the bottom and the outlet at the top, a cold discomfort zone near the inlet extends along the primary airflow path. However, secondary airflow currents mitigate this effect by redistributing cool air, enhancing thermal comfort in other parts of the room.

**Fig. 28:**
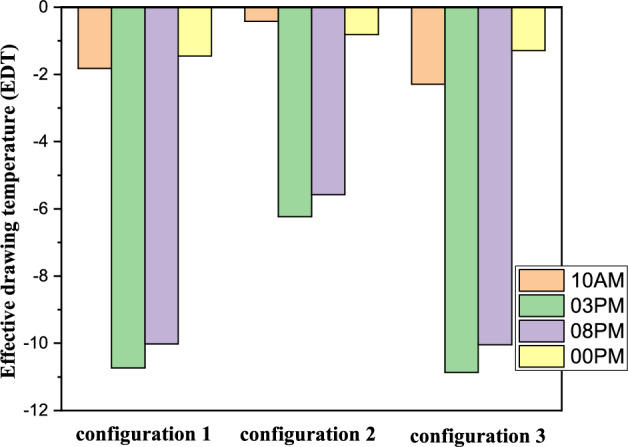
Histogram of average Effective Draft Temperature values (EDT) for different configurations.

This analysis highlights the influence of inlet and outlet configurations on thermal comfort zones, emphasizing the role of secondary airflow currents in mitigating cold discomfort. It also identifies critical areas within the room where air circulation patterns, shaped by inlet and outlet placement, significantly impact occupant comfort. Histogram 28 (Figure 28) compares the average EDT values for the selected configurations at different times of the day. Positive EDT values above +1.1 indicate uncomfortably warm conditions, while values below −1.7 indicate uncomfortably cool conditions. All configurations exhibit elevated EDT values (indicating cold discomfort) at 3:00 pm and 8:00 pm, whereas 10:00 am and midnight show lower values. Notably, Configuration 2 demonstrates lower average EDT values compared to Configurations 1 and 3, attributed to its ceiling openings that restrict airflow across the entire room. While this results in lower average EDT values, it may not fully capture the thermal comfort distribution throughout the space, underscoring the need for localized analysis.

**Fig. 29:**
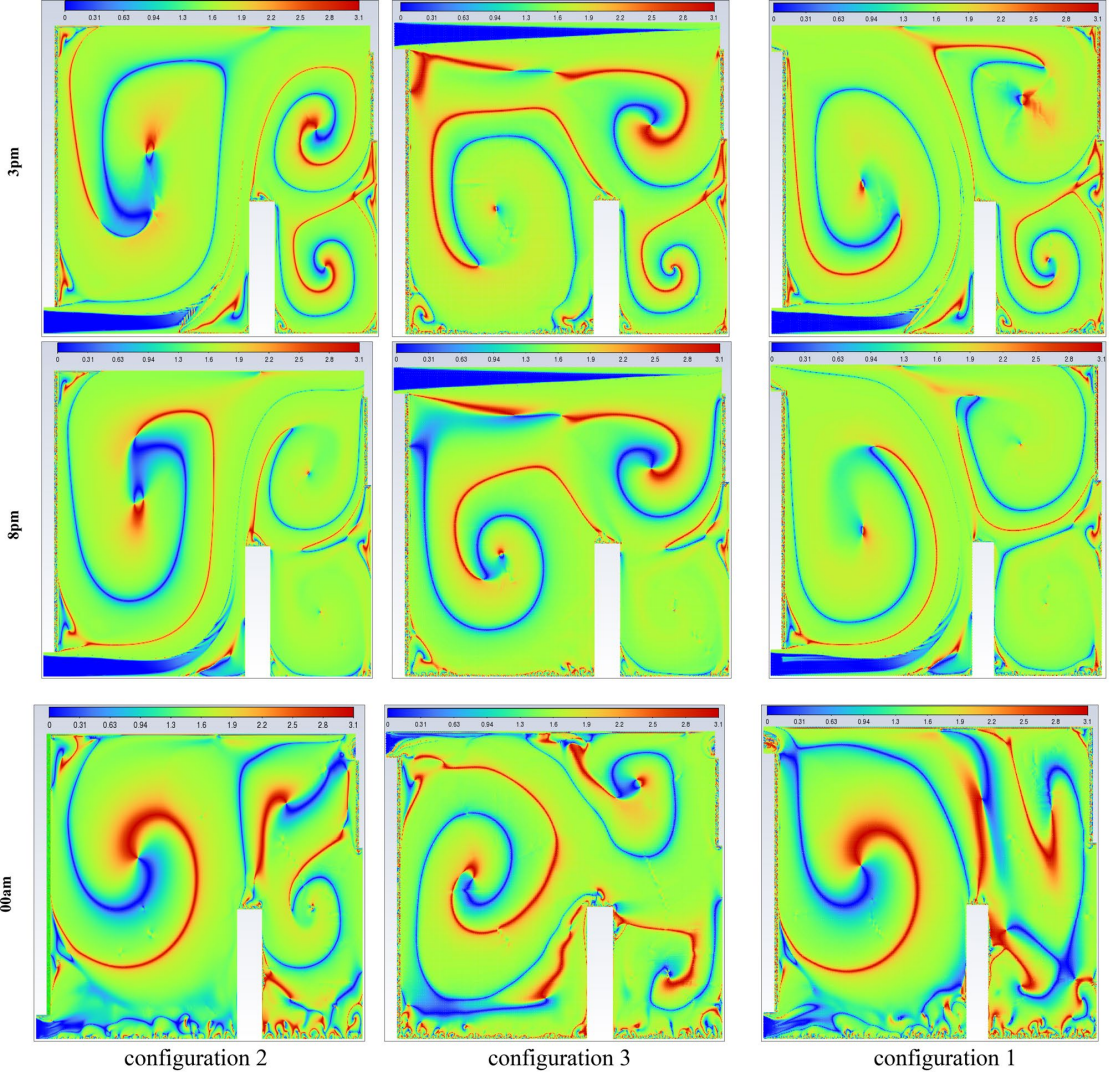
Contours of the synergy at different hours for all configurations.

This study represents a groundbreaking application of synergy principles to building dynamics, establishing a significant precedent in the field. Field synergy, which describes convective heat transfer driven by the scalar product of velocity and temperature gradient vectors, provides a robust framework for understanding how the alignment of these vectors enhances heat transfer efficiency. This concept is particularly valuable for identifying and explaining localized improvements in heat transfer mechanisms. While previous studies have often focused on average synergy angles across large computational domains, relying solely on velocity profiles fails to capture the intricate, localized thermal dynamics critical for a comprehensive understanding of heat transfer processes. The primary objective of this study is to calculate the synergy angle—a key metric directly linked to heat transfer efficiency—across various pre-defined room configurations. This involves analyzing shifts in thermal performance by examining changes in local velocity fields and temperature gradients through the lens of field synergy principles. Figure 29 presents the synergy angle profiles at different times of the day for all configurations, highlighting the impact of secondary airflows on heat transfer dynamics. The analysis of these profiles reveals distinct patterns: Configurations 1 and 3, which feature non-opposing placements for the air inlet and outlet, exhibit higher synergy levels compared to Configuration 2. This increased synergy is attributed to the presence of multiple secondary airflow paths, which facilitate more effective heat transfer beyond the room boundaries. These findings underscore the pivotal role of secondary flows in enhancing heat transfer, emphasizing their significance in optimizing indoor thermal regulation and overall building performance.

#### Direction test

In this section, a comparative study of the various wind directions (eastern, western, northern, southern) and their effects on the chosen formation 1 was presented. This study aimed to identify the most favorable wind direction and speed for the investigated area in order to position the air inlet and outlet along appropriate airflow trajectories, thereby enhancing indoor thermal comfort.

**Fig. 30:**
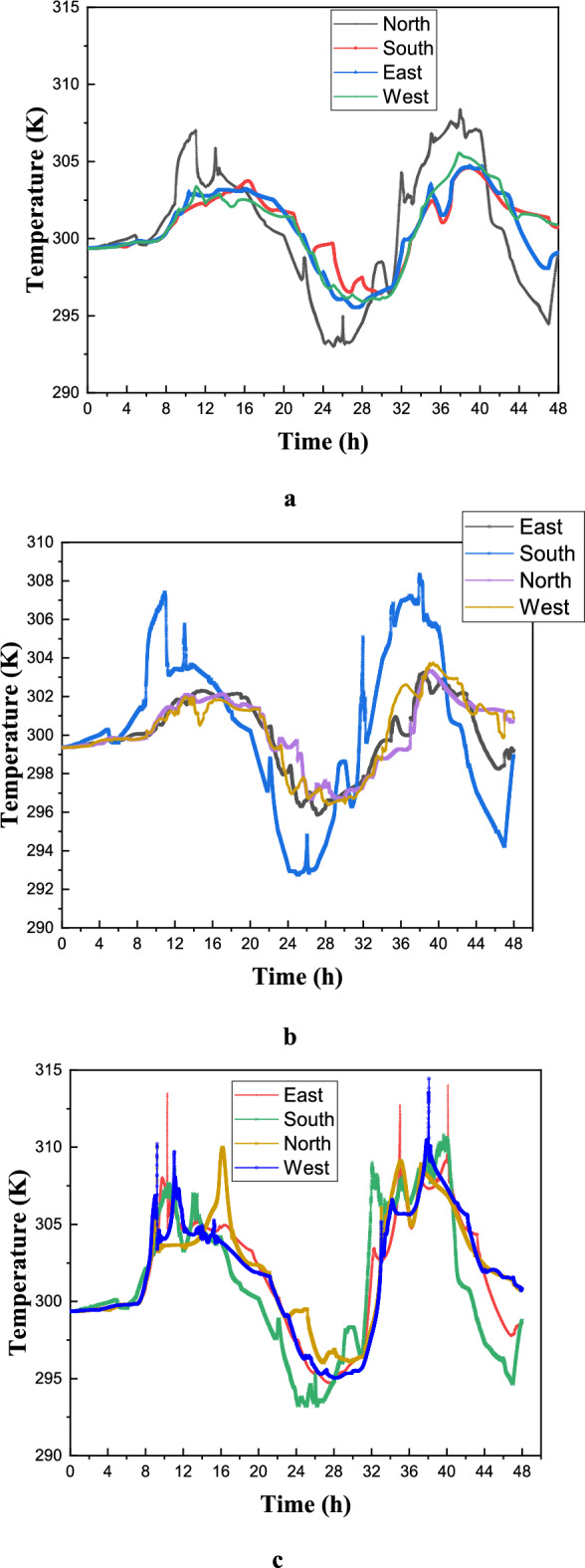
Distribution of air temperature inside the room over time (days in July) (**a**): Average temperature in the room; (**b**): Position A; (**c**): Position B.

Figures 30(a) illustrate the temporal distribution of average air temperatures inside the room for Configuration 1 under varying air input directions (east, west, north, and south) at specific locations. The analysis reveals that while wall temperatures remain relatively uniform across all cases, reflecting general air temperature trends influenced by wind direction, absolute uniformity is not fully achieved. Notably, northerly winds lead to an increase in average room air temperature, whereas easterly, westerly, and southerly winds contribute to a significant reduction in indoor temperatures. Among these, westerly winds produce the most substantial cooling effect, enhancing thermal comfort more effectively than the other orientations. The effects of easterly and southerly winds are relatively similar, though spatial variability exists. To further assess occupant comfort, temperature measurements were taken at two distinct points in the room: one to the right and one to the left of the occupant. Figure 30 (a,b) indicate that southerly winds may negatively impact comfort on the left side (point A, closest to the air inlet), while the right side experiences relatively uniform conditions across all wind directions. This spatial variation highlights the need to consider both air intake direction and occupant positioning to optimize thermal comfort and ventilation efficiency. The directional airflow study in Figures 29 and 30 provides valuable insights into how wind orientation influences indoor thermal conditions, with several practical implications for building design and HVAC optimization. The pronounced cooling effect of westerly winds suggests that architects in warm climates should prioritize west-facing ventilation openings to enhance passive cooling, particularly during peak afternoon heat. Conversely, the warming effect of northerly winds indicates that north-facing air intakes could assist with passive heating in colder regions, though seasonal adjustments may be necessary to maintain comfort year-round. The spatial temperature differences between points A and B demonstrate that even when wall temperatures remain uniform, localized comfort can vary significantly based on occupant positioning relative to airflow. This is especially relevant for spaces like offices and classrooms, where occupants remain stationary for extended periods. The similar performance of easterly and southerly winds provides flexibility in ventilation design, particularly in constrained urban environments where ideal orientations may not always be feasible. For retrofit applications, the data suggests that modifying existing ventilation pathways to replicate the cooling effects of westerly winds could significantly enhance thermal performance without requiring major structural modifications.

**Fig. 31:**
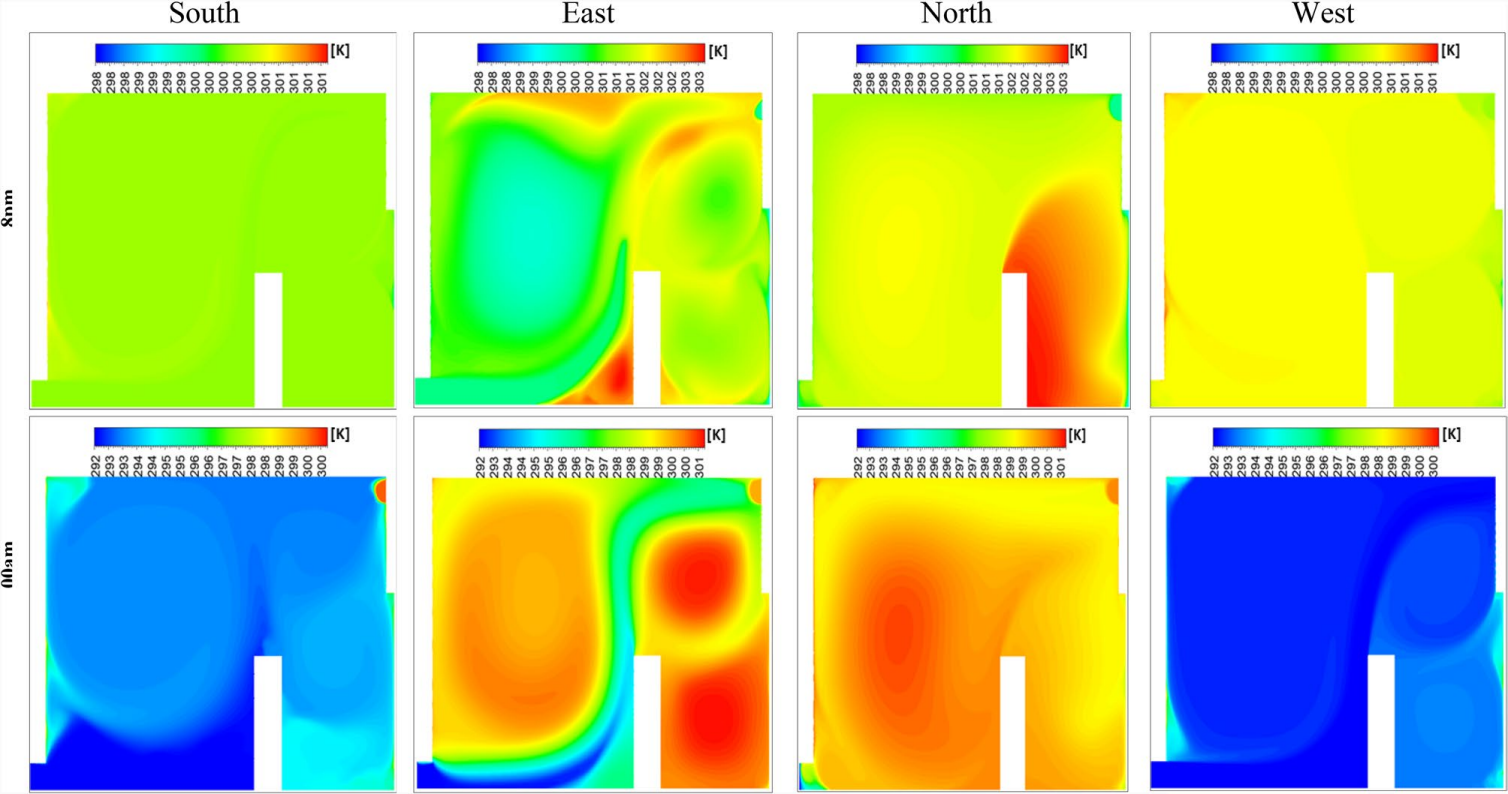
Contours of the isotherms for all directions at several different hours for configuration 1.

Figure 31 presents the isotherm profiles for various wind directions (east, west, north, and south) at two distinct times of the day, highlighting significant differences in temperature distributions influenced by wind patterns. For the south direction, temperatures of 300 K were recorded during the first hour, decreasing to 292 K in the second hour. Notably, the lower right side of the room registered 295 K, while the rest of the room maintained a temperature of 294 K. In contrast, the east and west directions exhibited consistent temperature values across both hours. During the first hour, both directions recorded 300 K for the wind trajectory inside the room and 302 K for the exterior. In the second hour, temperatures inside the room were 293 K, with values ranging between 292 K and 300 K in other areas. The north direction showed more variability, with temperatures of 303 K on the user’s right side and 301 K elsewhere during the first hour, decreasing to 298 K in the second hour. This detailed analysis of isotherm profiles for different wind directions provides valuable insights into the thermal dynamics influenced by wind patterns. Understanding these variations is essential for optimizing thermal comfort strategies and highlights the critical role of wind direction in the design and evaluation of room ventilation and temperature control systems.

**Fig. 32:**
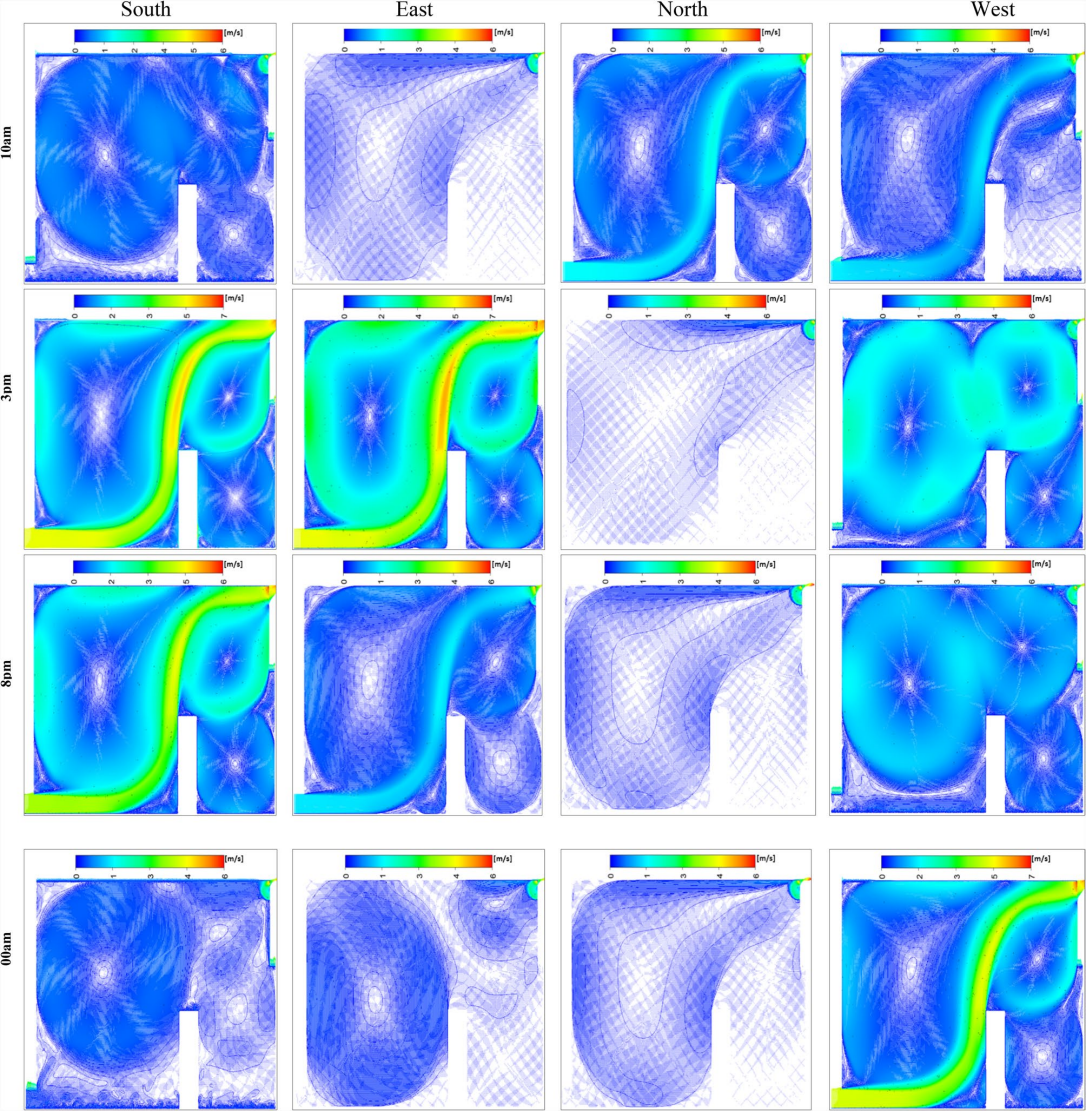
Contours of the velocity for all directions at several different hours for configuration 1.

In the context of identifying the most suitable wind direction for room ventilation, clear visualization and interpretation of airflow streamlines are essential for accurately assessing indoor air distribution and thermal performance. Figure 32 presents the intricate distribution of airspeed for all studied wind directions (east, west, north, and south) at various hours of the day. Notably, the first wind speed observation at 10 a.m. indicates generally low wind speeds across all scenarios. Specifically, the north direction records values close to 2 m/s, while the remaining directions experience light winds ranging between 0 and 1 m/s.

**Fig. 33:**
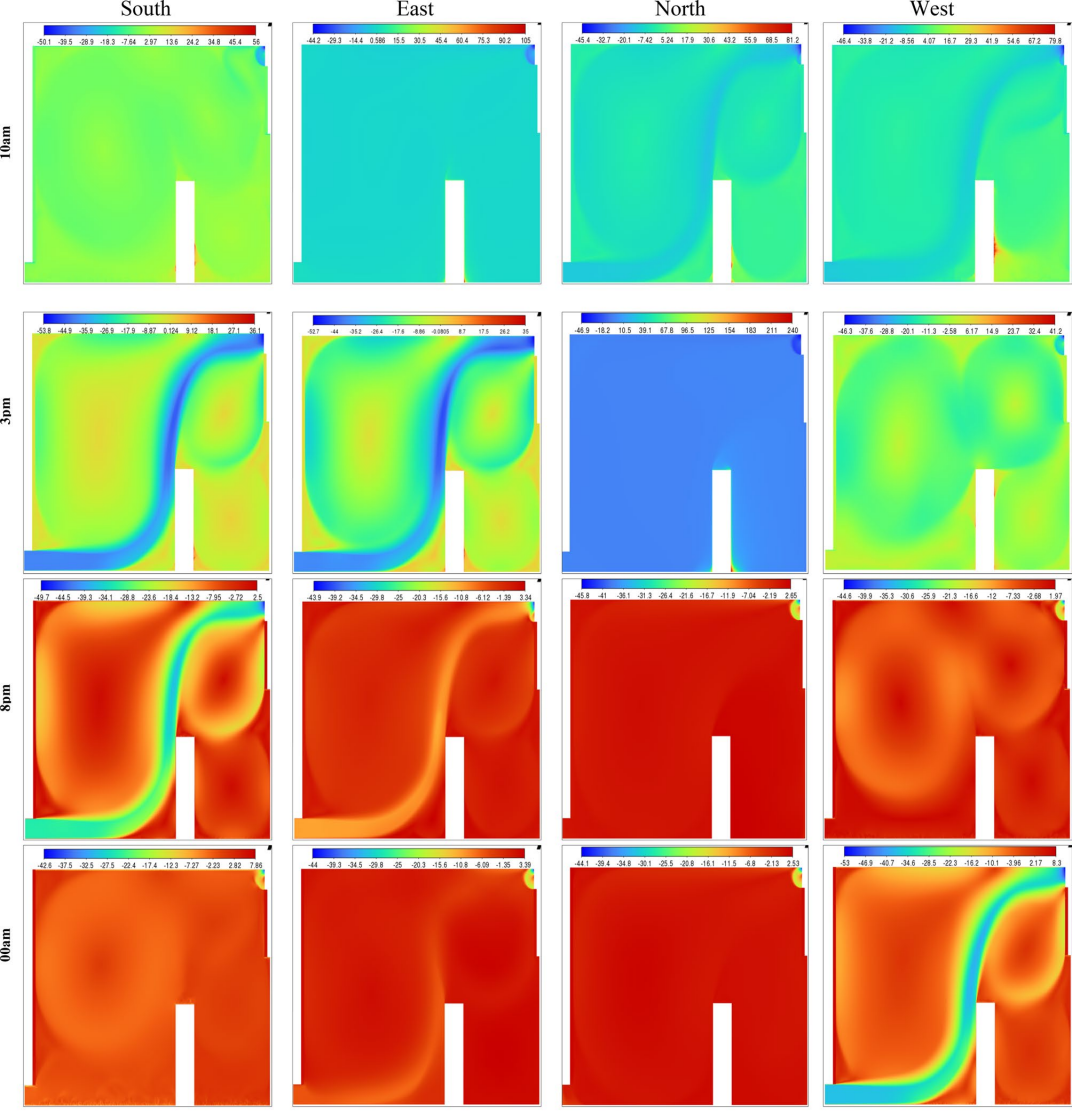
Contours of the EDT for all directions at several different hours for configuration 1.

**Fig. 34:**
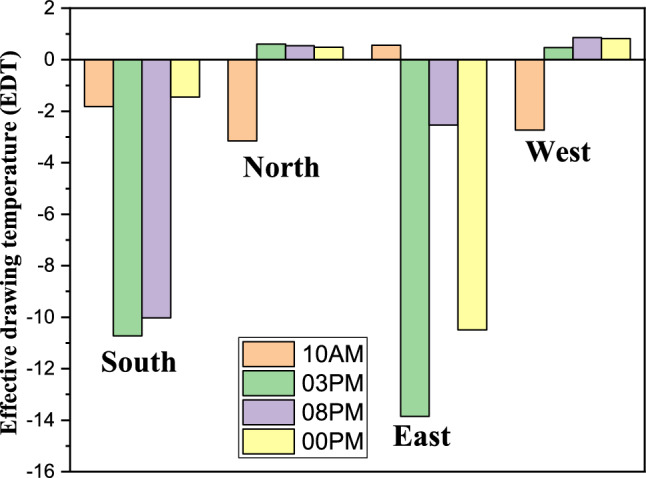
Histogram of average EDT for all directions at several different hours for configuration 1.

Throughout the day, the north-facing ventilation consistently maintains low wind speeds, ranging between 0 and 1 m/s. In contrast, the south-facing direction registers a wind speed of 5 m/s along the wind trajectory inside the room at 3:00 pm and 8:00 pm, with approximately 2 m/s at the room’s center. Similarly, the east-facing direction achieves a wind speed of 5 m/s along the wind trajectory, though other areas exhibit lower speeds. The west-facing direction follows a comparable trend in the afternoon and evening, while all directions show reduced wind speeds during early night hours, with the west direction maintaining slightly higher levels. This detailed analysis of wind speed variations provides critical insights into airflow dynamics, essential for optimizing wind-based room ventilation strategies. To identify the most favorable wind direction for room ventilation, the Effective Draft Temperature (EDT) index was employed as the primary metric for evaluating occupant thermal comfort under different wind orientations (east, west, north, and south). The Effective Draft Temperature (EDT) index, which depends on air temperature and speed, is highly sensitive to variations in these parameters. Figure 33 visualizes the comfort zones for each wind direction applied to the room’s air intake route, displaying EDT contours at various times of day and night. The results indicate that certain zones along the wind trajectories consistently exhibit discomfort, while central areas of the room generally maintain thermal comfort. At 10:00 am, all directions achieve thermal comfort. However, by 3:00 pm, the east and south directions show significant deviations from comfort, with EDT values approaching −28 for the south and +28 for the east, indicating discomfort. This trend continues at 8:00 pm for both directions. In contrast, the north and west directions maintain EDT values closer to the comfort range (between −7 and +10) throughout the day. After midnight, all wind directions move outside the defined thermal comfort range; however, the north-facing direction remains closer to the acceptable EDT limits over the 24-hour cycle, indicating comparatively more stable comfort performance. Consequently, the north-facing direction is identified as the most favorable for achieving consistent thermal comfort. Histogram 34 (Figure 34) compares the average EDT values for Configuration 1 across different wind directions (east, west, north, and south) at various times of day. EDT values between −1.7 and +1.1 are considered ideal for occupant comfort, with values outside this range indicating discomfort. At 10:00 am, all wind directions yield values close to the comfort range. However, at 3:00 pm, the south and east directions exhibit large negative values, indicating cold discomfort. In contrast, the north and west directions maintain values near the comfort range throughout the day. Although the EDT values for the east direction decrease at 8:00 pm, the north-facing direction consistently maintains values closer to the thermal comfort range, indicating its more stable and favorable performance under the investigated conditions.

### Economic viability of natural ventilation strategies

While various studies highlight the potential energy savings associated with natural ventilation strategies, their economic returns remain a subject of debate. Akermi Faouzi et al^58^.demonstrated that natural ventilation, particularly cross-ventilation, effectively enhances indoor thermal comfort and reduces energy consumption in historic residential buildings during warmer seasons. Similarly, Ahmad Walid et al^59^. found that cross-ventilation strategies could achieve energy savings of up to 65.6% for counter-cross ventilation and 18.7% for attached wind traps, significantly lowering cooling demand and associated costs. Despite these promising findings, the economic benefits of natural ventilation are not always directly proportional to the savings achieved. Factors such as initial design modifications, structural adaptations, and climate variations can impact the overall financial feasibility. While natural ventilation offers long-term sustainability advantages, its economic returns may not always align with the energy reductions it provides, requiring further cost-benefit analysis to assess its true financial impact.

## Conclusion

This study numerically investigated the combined use of phase change materials (PCMs) and natural ventilation to improve thermal comfort in buildings under the arid summer climate of Oum El Bouaghi, Algeria. A comparative CFD-based parametric analysis was conducted to evaluate four PCM candidates and multiple ventilation configurations under cooling-dominant July conditions.

Among the investigated materials, n-hexadecane provided the strongest instantaneous temperature reduction during peak solar hours due to rapid latent heat absorption. However, n-octadecane exhibited more stable and prolonged thermal regulation, maintaining interior surface temperatures near its melting range (301–302 K) throughout the daily cycle. Based on supplier unit material prices per kilogram, n-octadecane also presents approximately 98% lower raw material cost than n-hexadecane, although this comparison does not represent full system or lifecycle cost. Increasing PCM thickness reduced daily integrated heat flux by up to 52% at 10–15 cm; however, diminishing returns were observed beyond this range, and thinner layers may offer a more practical engineering compromise.

Regarding ventilation, the configuration with a bottom inlet and top outlet on opposite walls achieved the most stable indoor conditions, reducing indoor air temperature by up to 2 K and improving airflow distribution. Westerly winds enhanced cooling performance, while north-facing ventilation maintained more stable Effective Draft Temperature (EDT) values. The application of EDT and the synergy angle provided additional insight into the interaction between airflow and heat transfer in defining comfort zones.

Overall, the results highlight the potential of integrating PCM-enhanced walls with climate-adaptive natural ventilation as a passive cooling strategy. Future work should extend the analysis to annual conditions and include experimental validation and comprehensive economic assessment.

## Data Availability

The datasets used and/or analysed during the current study available from the corresponding author on reasonable request.

## List of symbols

c_p_: Specific heat capacity (kJ ${kg}^{- 1}$ ${K }^{- 1}$)
g: Gravitational acceleration (m $${s}^{- 2}$$)
h: Transfer coefficient (W $${m}^{-2}{K }^{- 1}$$)
${\mathrm{h}}_{0} {\mathrm{h}}_{\mathrm{out}}$: Border conditions transfer coefficient (W ${m}^{-2}{K }^{- 1}$)
H: Specific enthalpy ($$Jk{g}^{-1}$$)
$${H}_{\mathrm{sens}}$$: Sensible enthalpy ($$Jk{g}^{-1}$$)
$${H}_{\mathrm{lat}}$$: Latent enthalpy ($$Jk{g}^{-1}$$)
k: Turbulent kinetic energy ${(m}^{2}$/${s}^{2}$)
P: Pressure (N $${\mathrm{m}}^{- 2}$$)
Pr: Prandtl number (1)
T: Temperature (K)
t: Time (s) (s)
u: Velocity (m $${s}^{- 1}$$)
$${\mathrm{u}}_{\mathrm{i}}$$$${\mathrm{u}}_{\mathrm{j}}$$: The velocity components in the direction of each spatial coordinate in a Cartesian coordinate system (m $${s}^{- 1}$$)
U: Velocity (m $${s}^{- 1}$$)
v: Velocity vector (m $${s}^{- 1}$$)
w: Wind speed (m $${s}^{- 1}$$)
X: Spatial direction
Y: Spatial direction
$${\mathrm{x}}_{\mathrm{i}}$$$${\mathrm{x}}_{J}$$: Spatial coordinates in cartesian coordinate system
β: Thermal expansion coefficient (${\mathrm{K}}^{- 1}$)
$$\uplambda$$: Thermal conductivity (W $${\mathrm{m}}^{- 1}{\mathrm{K}}^{- 1}$$)
ρ: Density (kg $${m}^{-3}$$)
$\upxi ,\upeta$: Phase field parameters used in PCM phase-change modeling
${\mathrm{e}}_{\mathrm{i}}$: Unit vector in the i-direction
θ: Synergy angle, representing the angle between velocity and temperature gradient vectors°
$\mu$: Molecular (dynamic) viscosity of the fluid Pa s
${\mu }_{t}$: Turbulent viscosity (Pa s)

## Subscripts

lat: latent
ref: reference
PCM: phase change material
sens: sensible
EDT: Effective draft temperature
